# A new basal ornithopod (Dinosauria: Ornithischia) from the Early Cretaceous of Texas

**DOI:** 10.1371/journal.pone.0207935

**Published:** 2019-03-12

**Authors:** Kate A. Andrzejewski, Dale A. Winkler, Louis L. Jacobs

**Affiliations:** Roy M. Huffington Department of Earth Sciences, Southern Methodist University, Dallas, Texas, United States America; George Washington Univ, UNITED STATES

## Abstract

Material from a minimum of twenty-nine individuals of a new ornithopod, represented by nearly every skeletal element, was recovered from the Proctor Lake locality in the Twin Mountains Formation (Aptian) of north-central Texas. This material includes various ontogenetic stages, providing insight into the growth patterns of this species. The new ornithopod, *Convolosaurus marri* gen. et sp. nov., is recovered outside of Iguanodontia, but forms a clade with Iguanodontia exclusive of *Hypsilophodon foxii*. The presence and morphology of four premaxillary teeth along with a combination of both basal and derived characters distinguish this taxon from all other ornithopods. Basal characters present in *C*. *marri* including the presence of premaxillary teeth, the shape of the dentary teeth, and position of the pterygoid wing on the quadrate, whereas the presence of opisthocoelous cervical vertebrae, large proximal caudal neural spines, and curved maxillary tooth roots suggest *C*. *marri* is more derived than 80% of the basal neornithischians included in this analysis.

## Introduction

**Fig 1:**
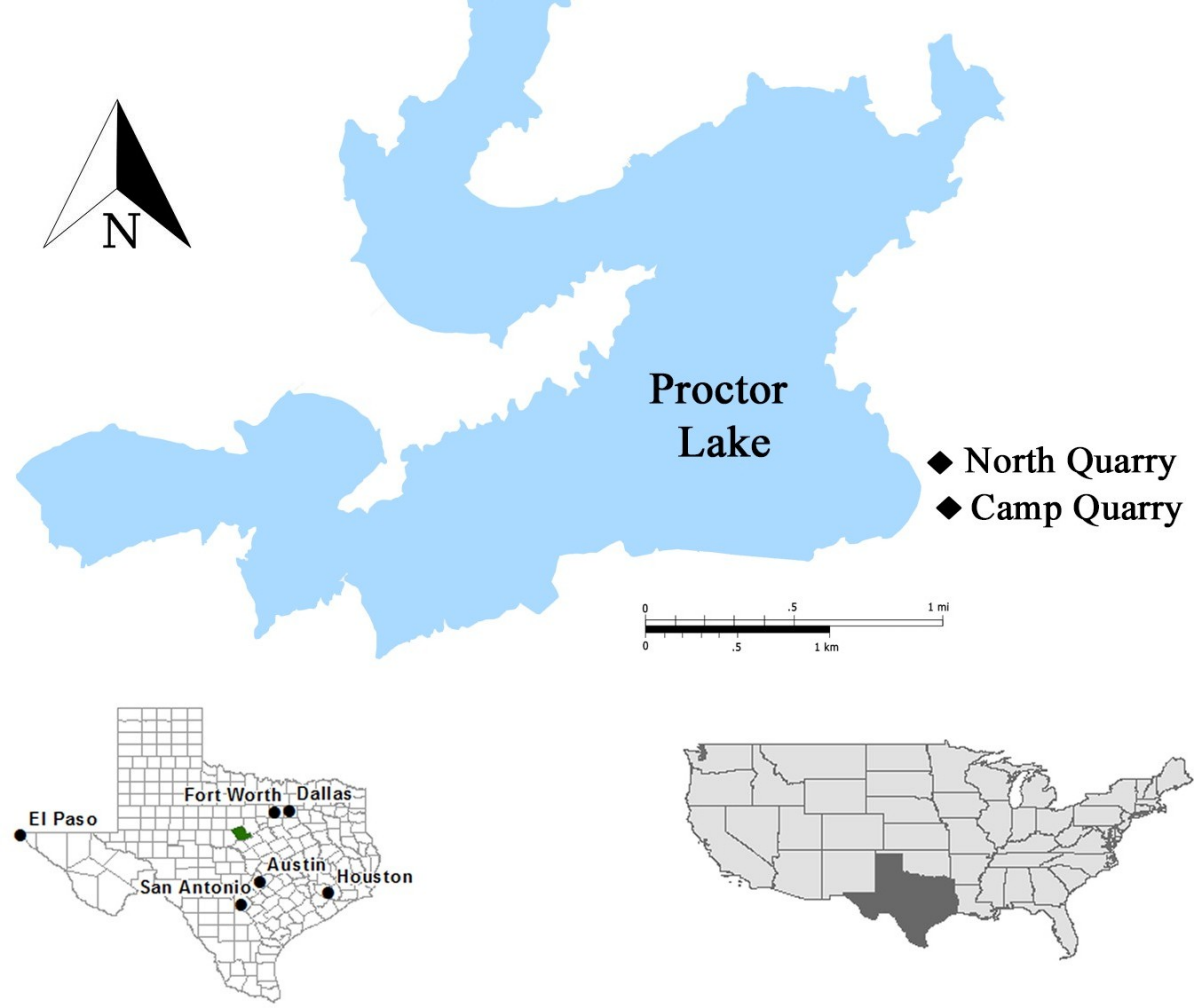
Map of study area. Map of Proctor Lake, Texas located in Comanche County (green) with marked locations of the two quarries (Camp Quarry and North Quarry) where ornithopod fossils were collected.

**Fig 2:**
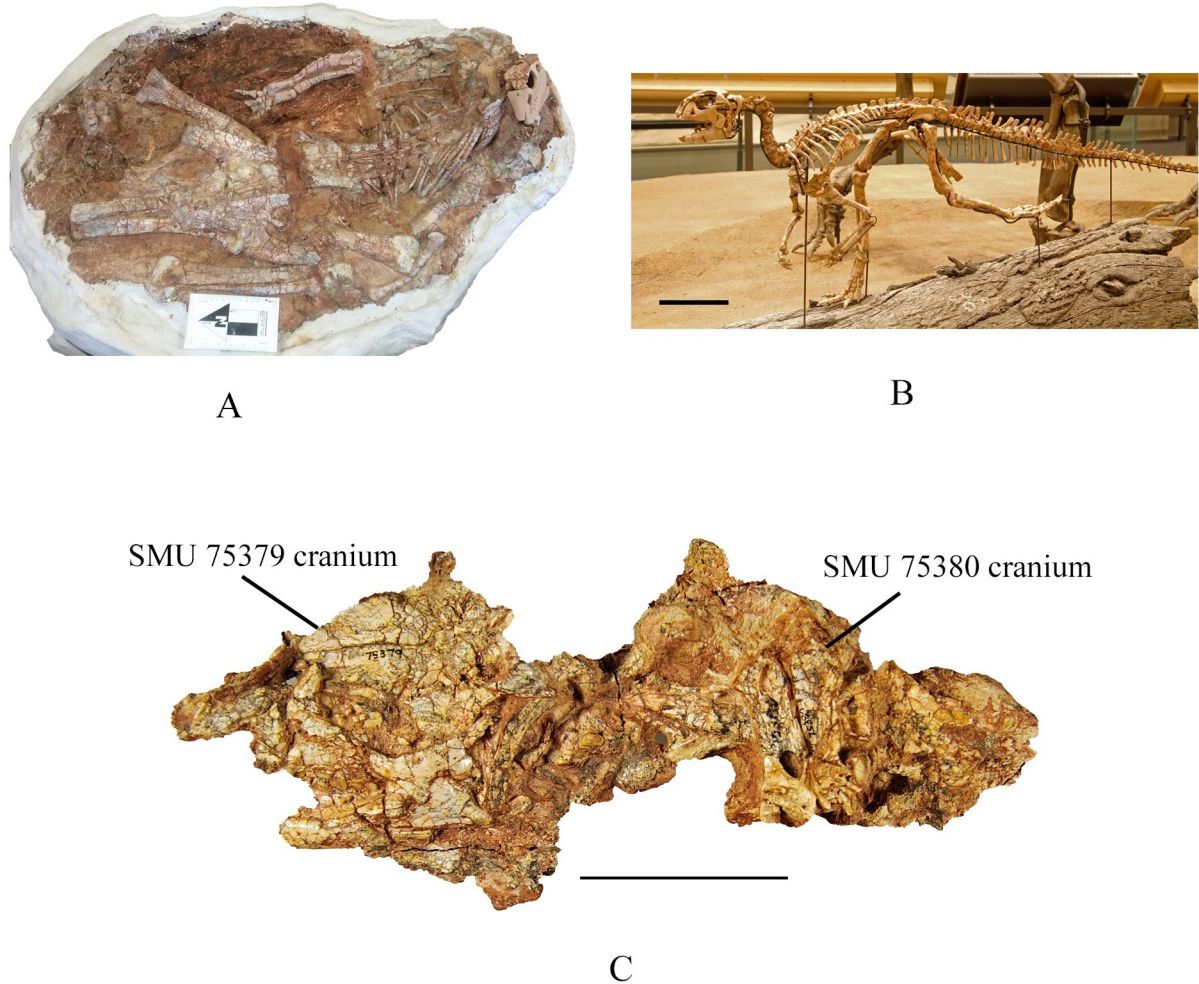
Articulated specimens. (A) SMU 70456, articulated subadult individual on display at the Proctor Lake Corps of Engineers Office. Scale arrow equals 10 cm. (B) Composite skeleton on display at the Perot Museum of Nature and Science. Scale bar equals 10 cm. (C) SMU 75379 and SMU 75380, partial articulated skeletons found stacked on one another. Scale bar equals 5 cm.

Abundant remains of a small ornithopod dinosaur were first discovered near Proctor Lake, Texas in May 1985 in sediments of the Lower Cretaceous Trinity Group (Fig 1). More dinosaur specimens have been recovered from this locality than from any other Early Cretaceous site in Texas. At least 29 individuals of a new ornithopod, including several larger articulated individuals and mass accumulations of partially articulated smaller individuals were recovered (Fig 2). Initial studies suggested that some mass accumulations of smaller individuals occur in depressions possibly reflecting a nesting site, although no egg shell has been recovered [1], and that these groupings may have represented precocious flocks. The purpose here is to diagnose and describe the Proctor Lake ornithopod and to determine its evolutionary position through phylogenetic analysis.

**Fig 3:**
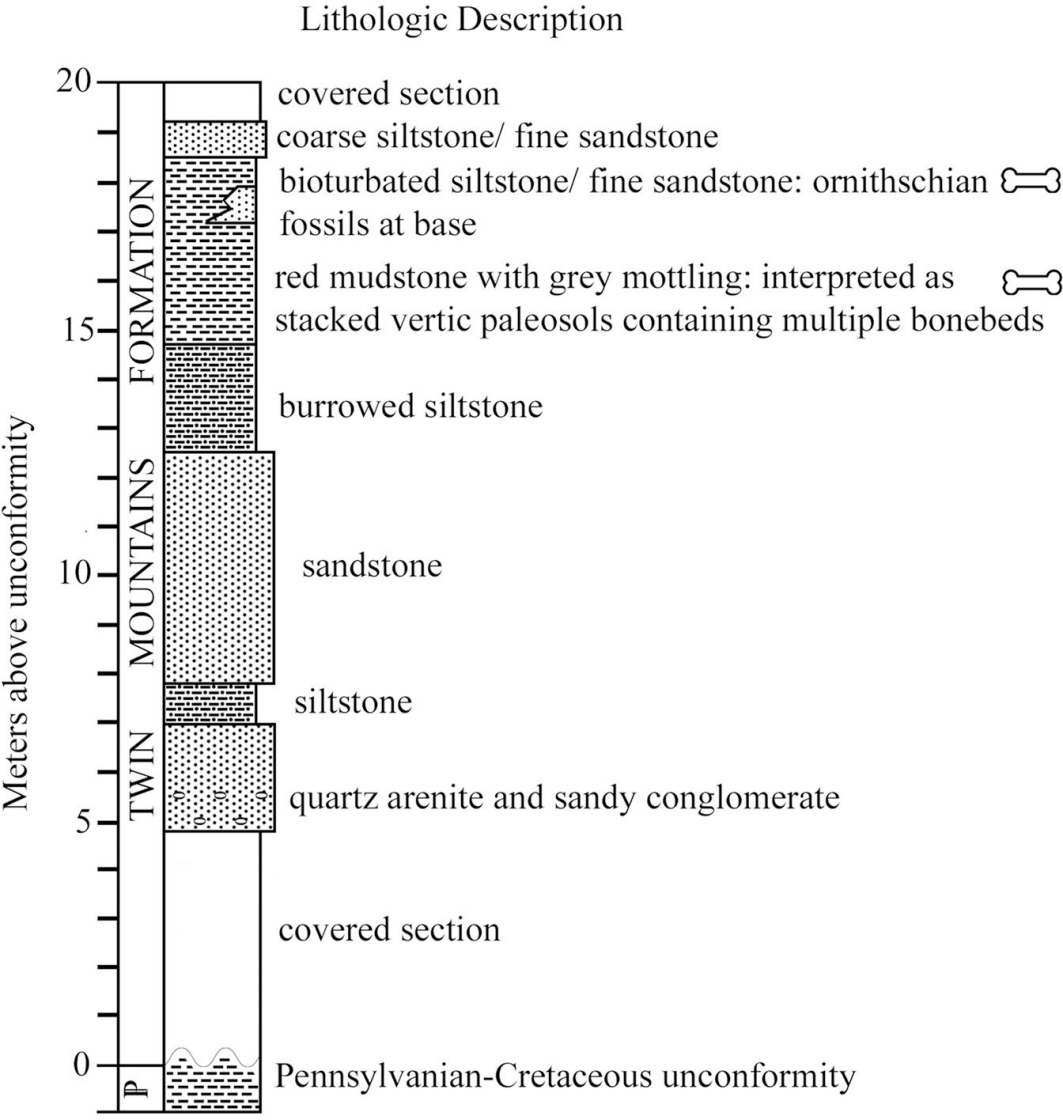
Modified stratigraphic column of Proctor Lake fossil locality. Stratigraphic section of Twin Mountains Formation exposed at Proctor Lake (modified from Winkler and Murry, 1989).

The Proctor Lake locality sits stratigraphically low in the Twin Mountains Formation, the lowest of the three formations in the Trinity Group in this area (Twin Mountains, Glen Rose, Paluxy). Although the age of the Twin Mountains Formation is not tightly constrained, its unconformable base may be Aptian (~ 125 Ma) or older, and the base of the overlying Glen Rose Formation is biostratigraphically near the Aptian-Albian boundary (~113 Ma) in the vicinity of Proctor Lake [2; 3; 4; 5]. The Twin Mountains sequence includes terrestrial and transitional marine facies representing the earliest Cretaceous transgressions upon the Texas craton [6], prior to the Albian Glen Rose transgression and the late Albian completion of the Western Interior Seaway. The basal portion of the Twin Mountains Formation consists primarily of conglomerates and sandstones and the upper portion comprises thinly interbedded sands and mudstones (Fig 3; [1]), interpreted as fluvial flood-basin facies. The fossil-bearing strata at Proctor Lake that produced the ornithopod described here are 2 m of reddish muds and sands from the lower portion of the Twin Mountains Formation [1; 3; 7]. The cross-sectional profile from the Proctor Lake fossil localities contains dark red (2.5 YR 3/4, 3/6) stacked B horizons, classified as well-developed calcic Vertisols. The clay rich B horizons contain prismatic peds that coarsen upwards through the profile. Pedogenic carbonate nodules were recovered in lower parts of the profile, but the calcareous matrix is highly reactive throughout the profile. Infill dikes ~6 cm in diameter cross cut the entire profile and vermicular mottles in diameter are found in the lower parts of the profile.

Vertebrate fossils occur throughout the Twin Mountains Formation [3]. Other archosaur taxa recovered with the Proctor Lake ornithopod include a crocodyliform, *Wannchampsus kirpachi* [8], and a single tooth of a dromaeosaur. The upper portion of the Twin Mountains contains a variety of fauna, including the crocodyliform *Paluxysuchus newmani* [9], sauropod remains at the Jones Ranch site [10; 11], the theropod *Acrocanthosaurus atokensis* [12], and the ornithopod *Tenontosaurus dossi* [13].

## Materials and methods

The material described is curated at the Shuler Museum of Paleontology at Southern Methodist University (SMU) Dallas, Texas except for the following specimens: SMU 70456 (on display at the Proctor Lake US Army Corps of Engineers Office in Proctor, Texas), SMU 74087, 74093, and 74104 (composite skeleton on display at the Perot Museum of Nature and Science in Dallas, Texas), and SMU 74663 (composite skeleton on display at the Fort Worth Museum of Science and History in Fort Worth, Texas). The fossil material currently consists of 488 specimens which were collected from 48 localities which are outlined in two quarries designated “Camp Quarry” and “North Quarry” along the southeast shores of Proctor Lake, Texas (Fig 1). Specimen localities are listed with referenced specimens in supplementary material and locality maps are housed with the specimens in the Shuler Museum of Paleontology collection at SMU. Permission to excavate the site was obtained from the Proctor Lake US Army Corps of Engineers Office.

The material described consists of articulated and disarticulated elements. The collection represents a minimum of 29 individuals of the same ornithopod taxon. This estimation is based on 19 complete and 26 fragmentary femora recovered with associated right and left femora conservatively estimated as one individual based on locality and size. Length, proximal width, and distal width were measured for 19 complete femora and used to create both linear and logarithmic regressions. The linear regressions were used to estimate the length of partial femora because these regressions produced tighter correlations than the logarithmic functions. The measured and estimated length of the 29 individuals was then plotted on a histogram to show size distribution.

Histological analyses of four femora (SMU 70447, SMU 74085, SMU 73569, SMU 72834) were conducted by creating cross sectional thin sections near the midshaft of the femora and analyzing bone microstructures with polarized light microscopes. Four fossil localities (BMQ, 3BS, 1B7, and 2DU) with clusters of individuals were analyzed for size distribution by plotting measured or estimated femora length. Each locality consisted of three or more semi-articulated individuals within an area of 2 m^2^ or less.

**Nomenclature Acts:** The electronic edition of this article conforms to the requirements of the amended International Code of Zoological Nomenclature, and hence the new names contained herein are available under that Code from the electronic edition of this article. This published work and the nomenclatural acts it contains have been registered in ZooBank, the online registration system for the ICZN. The Zoobank LSIDs (Life Science Identifiers) can be resolved and the associated information viewed through any standard web browser by appending the LSID to the prefix “http://zoobank.org/”. The LSID for this publication is: urn:lsid:zoobank.org:pub:52E5580B-4912-4755-A604-9BD71DE4D2A5. The electronic version of this work was published in a journal with an ISSN, and has been archived and is available from the following digital repositories: Pub Med Central and LOCK.

### Systematic Paleontology

DINOSAURIA Owen, 1842

ORNITHISCHIA Seeley, 1887

NEORNITHISCHIA Cooper, 1985

CERAPODA Sereno, 1986

ORNITHOPODA Marsh, 1881

*Convolosaurus marri* gen. et sp. nov. urn:lsid:zoobank.org:act:468990B8-F35E-4165-8065-9FE0DA581D8A

**Etymology:** The generic name *Convolosaurus* translates from Latin meaning “flocking lizard” referring to clusters of juvenile specimens. The species name *marri* is in honor of Dr. Ray H. Marr who produced the Society of Vertebrate Paleontology videos *“We are SVP”* and *“About the SVP Logo”* posted on the SVP website (vertpaleo.org), and who is a strong proponent of students at Southern Methodist University (SMU).

**Holotype:** SMU 72834, a skull and partial articulated skeleton with 9 cervical vertebrae; 15 dorsal vertebrae; 6 sacral vertebrae; 23 caudal vertebrae; right and partial left scapula; right and partial left coracoids; left and partial right humeri; left ulna; left radius; partial left manus; articulated pelvis including the left and right ilia, proximal left and right ischia, partial prepubic rods; proximal and distal ends of the left and right femora and the mid-part of the left shaft; proximal left and right tibiae; and proximal left fibula. The type specimen, SMU 72834, is the largest individual in the sample measuring approximately 2.5–3 m in length; however, this skeleton does not represent a full grown adult, thus the adult size of this species in unknown.

**Diagnosis:** The presence of four premaxillary teeth with proximodistally oriented sulcus on the buccal surface distinguishes *Convolosaurus marri* gen. et sp. nov. from all other ornithopods. Further, it can be distinguished from other basal ornithopods by a unique combination of primitive and derived character states. Primitive character states include the presence of premaxillary teeth and two supraorbitals that extend across the entire orbit. Derived character states include: curved maxillary tooth roots; opisthocoelous cervical vertebrae; sacral neural spines twice the height of the sacral centra; proximal caudal neural spines 1.5 times the height of the centrum; expanded ischial ‘foot’; shallow intercondylar groove on the anterior surface of the femur; and a laterally compressed prepubic process.

**Referred Specimens:** SMU 70444, partial skull; SMU 70456, articulated skeleton; SMU 70534, articulated left hindlimb; SMU 70635, partial right maxilla; SMU 71492, caudal vertebra; SMU 71504, right paroccipital process; SMU 71510, right surangular; SMU 71631, right quadratojugal; SMU 71690, left astragalus; SMU 71818, left radius; SMU 71821, left ulna; SMU 71854, right scapula; SMU 72054, left manus; SMU 72316, partial skull, articulated pelvic girdle and vertebral column, partial left hindlimb; SMU 72534, left calcaneum; SMU 72541, left distal femur; SMU 73170, articulated left pes; SMU 73171, right pes; SMU 74087, partial skull; SMU 74093, right femur and tibia; SMU 74104, partial left pubis; SMU 74119, distal left ischium; SMU 74124, left scapula; SMU 74131, cervical vertebrae; SMU 74576, articulated caudal vertebrae with ossified tendons; SMU 74663, skull and partial skeleton; SMU 74664, left and right scapulae; SMU 74665, partial right hindlimb; SMU 74670, articulated caudal vertebrae with ossified tendons; SMU 74678, partial skull; SMU 74749, skull and partial skeleton; SMU 75379, partial skull; SMU 75380, right premaxilla; SMU 75564, partial skeleton; SMU 75621, axis and third cervical vertebra; SMU 75636, partial left pubis; SMU 77617, partial skeleton; SMU 77638, partial skeleton. Although these specimens vary in size, they are taken to represent a single species because of consistency in growth rate based on femora measurements and similar overall skeletal morphology.

**Locality:** Proctor Lake (SMU 001), Comanche County, Texas, Twin Mountains Formation, Early Cretaceous (Aptian).

### Description

#### Skull

**Fig 4:**
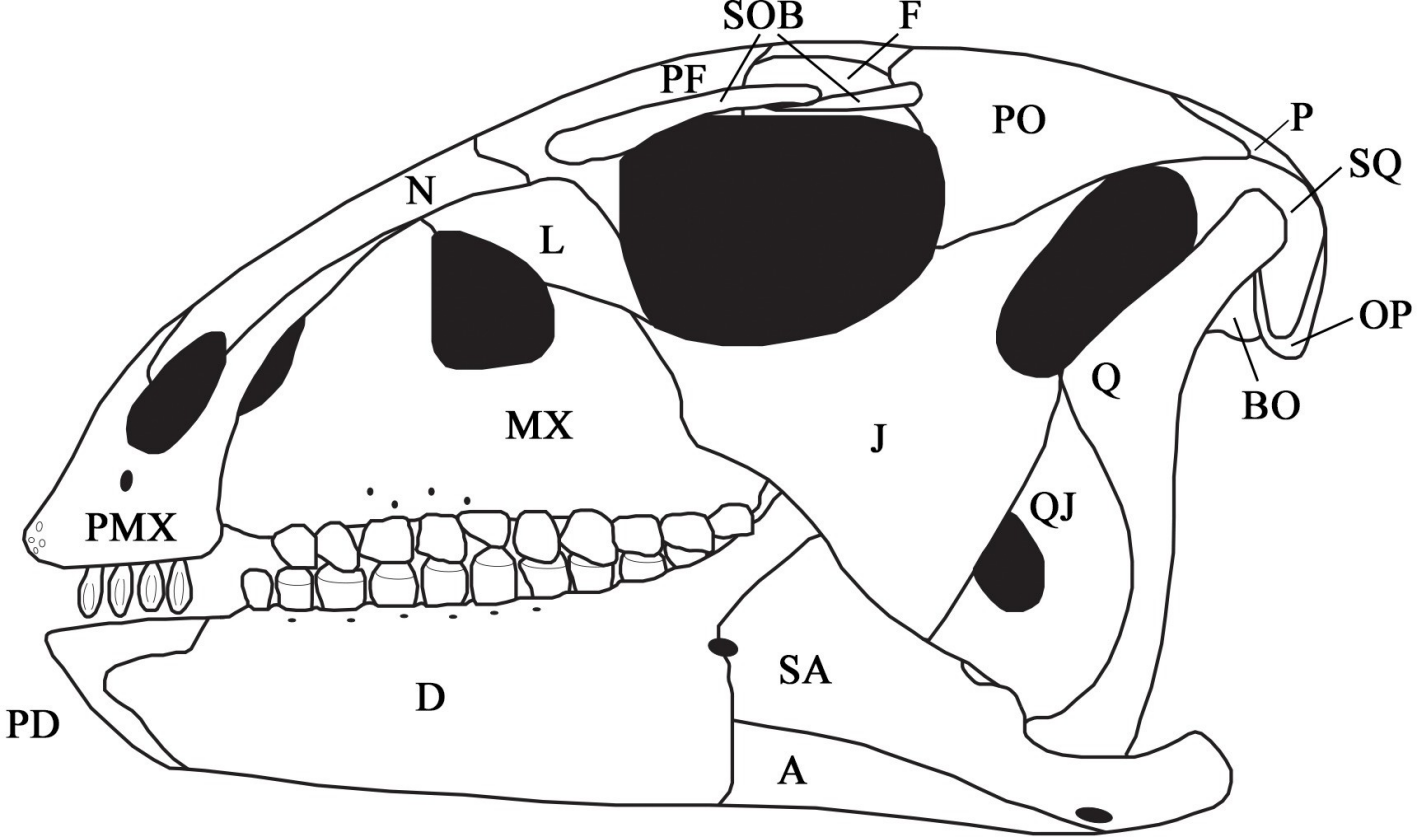
Skull reconstruction. Skull reconstruction of *C*. *marri* based on available specimens. Abbreviations: A-articular, BO-basioccipital, D-dentary, F-frontal, J-jugal, L-lacrimal, MX-maxilla, N-nasal, OP-opisthotic, P-parietal, PD-predentary, PF-prefrontal, PMX-premaxilla, PO-postorbital, Q-quadrate, QJ-quadratojugal, SA-surangular, SOB-supraorbital, SQ-squamosal.

Seven partially articulated specimens and four disarticulated elements were used for skull description. SMU 72834 is the largest and presumably most mature skull with a dentary length 2.75 times larger than the smallest complete dentary, SMU 70444. The remaining specimens are also significantly smaller and presumably represent younger individuals. Elements that are clearly preserved are described below. Elements that are not preserved or are difficult to interpret based on preservation include the ectopterygoid, parasphenoid, laterosphenoid, coronoid, articular, palatine, vomer, and prearticular. Fig 4 represents a skull reconstruction based on the available specimens.

#### Premaxilla

**Fig 5:**
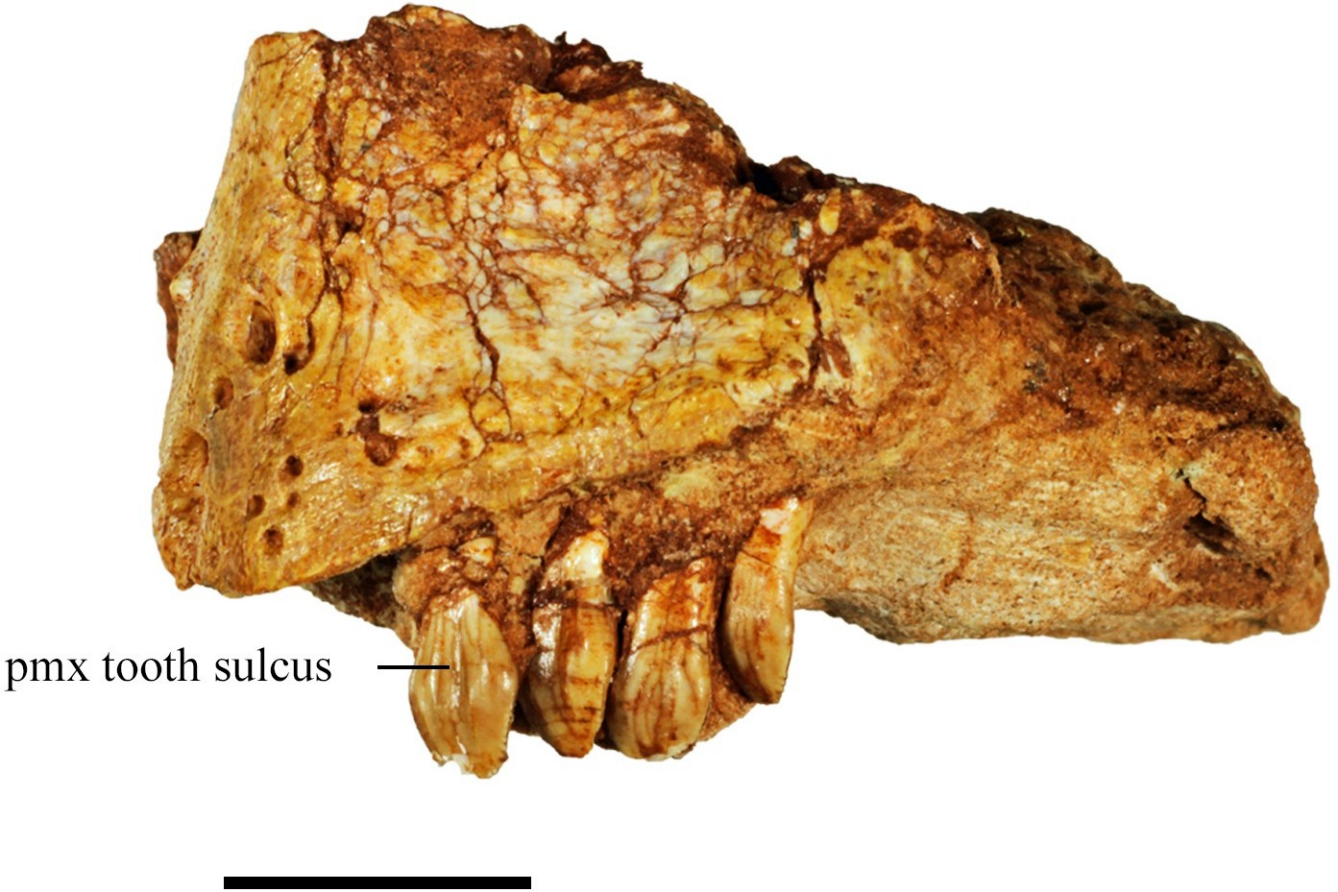
Premaxilla from specimen SMU 72316. Left premaxilla (SMU 72316) in lateral view containing a rugose anterodorsal surface and four premaxillary teeth displaying basal-apical oriented sulcus on the premaxillary teeth. Scale bar equals 1 cm.

**Fig 6:**
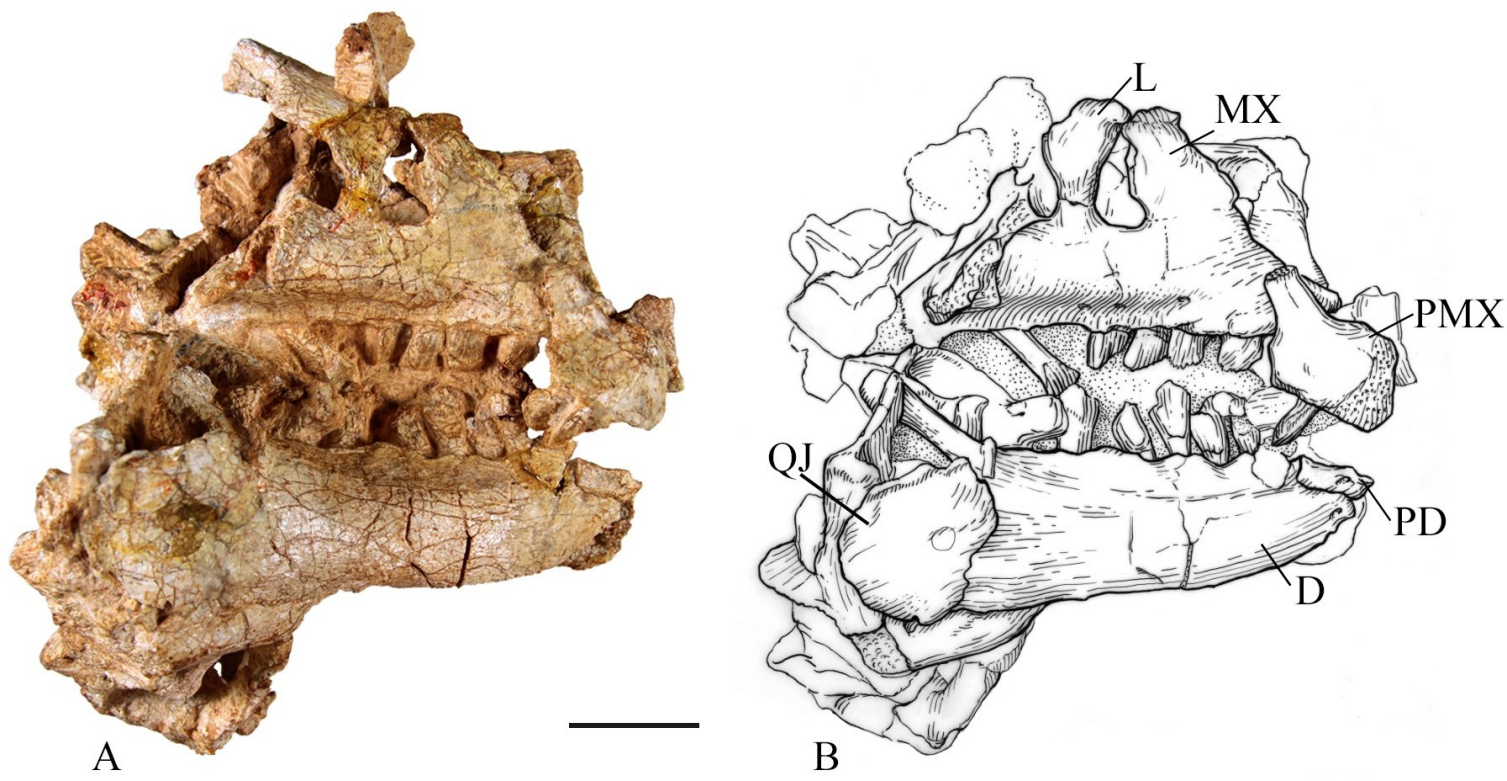
SMU 72834 anterior skull. (A) SMU 72834, anterior skull in right lateral view. (B) Illustration of SMU 72834, in right lateral view (David Baker). Abbreviations: D-dentary, L-lacrimal, MX-maxilla, PMX-premaxilla, PD-predentary, QJ-quadratojugal. Scale bar equals 5 cm.

SMU 72316 and the type specimen SMU 72834 contain partial premaxillae that are not fused to their pair in the midline (Figs 5 and 6). The premaxilla forms the anteroventral portion of the narial opening. The dorsal processes of the premaxillae overlap and wedge between the anterior ends of the nasals. The lateral surface of the oral margin is slightly flared and the ventral margin of the premaxilla is ventrally deflected compared to the maxillary tooth row. A fossa is present on the medial surface of the posteroventral corner of the premaxilla which receives the anterolateral process of the maxilla. The posterolateral process of the premaxilla does not contact the lacrimals; however, the left side of SMU 74749 preserves the maxilla rising as a thin sheet that overlaps the posterolateral process of the premaxilla and can contact the nasals making character 7 in the character matrix a problematic character to score.

The anterodorsal surface of the premaxilla is rugose, containing three to five small foramina. This region of the premaxilla likely supported a rhamphotheca [14]. The lateral surface of the premaxilla from SMU 72316 and SMU 75380 contains a foramen that is larger relative to the small foramina on the anterodorsal surface. This larger foramen pierces the premaxilla and is located approximately 0.5 cm below the anteroventral corner of the nasal opening. The anterior tip of the premaxilla contains a short edentulous region and there is a diastema measuring the width of one tooth between the maxillary and premaxillary tooth row. The premaxillae from SMU 72316, SMU 70444, SMU 74087, and SMU 75380 contain four premaxillary teeth. These specimens vary in size with the anterior-posterior tooth row length of the premaxilla ranging from 11 mm to 25 mm; therefore, it is unlikely that the number of premaxillary teeth changed ontogenetically.

#### Maxilla

**Fig 7:**
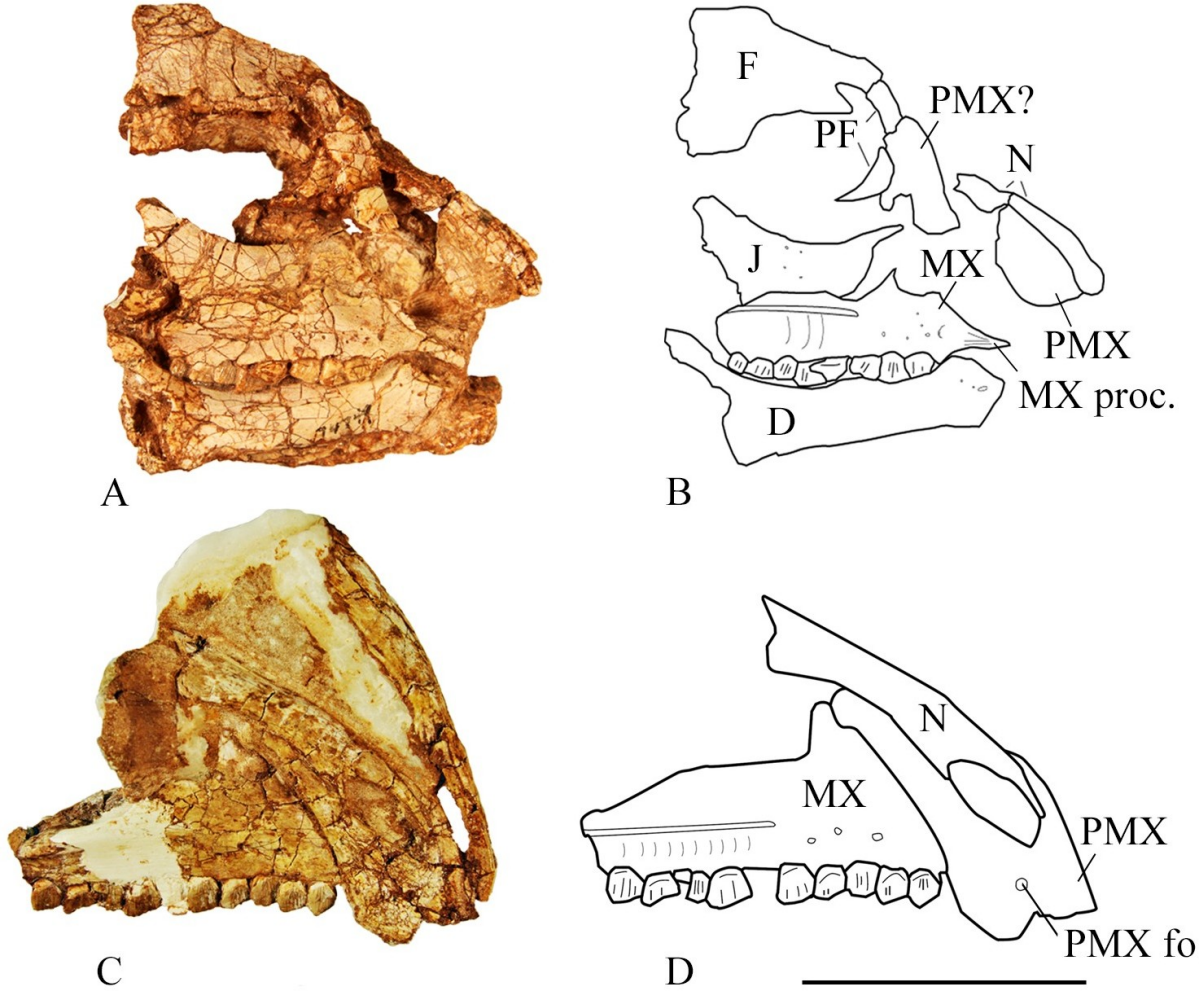
SMU 74749 and SMU 72316 cranial material. (A) SMU 74749, partial cranium in right lateral view. (B) Outline drawing of identifiable bone contacts on the right side of SMU 74749 partial cranium. (C) SMU 72316, partial cranium in right lateral view. (D). Outline drawing of identifiable bone contacts on the right side of SMU 72316 partial cranium. Abbreviations: D-dentary, F-frontal, J-jugal, MX-maxilla, MX proc.-anterolateral process of the maxilla, N-nasal, PF-prefrontal, PMX-premaxilla, PMX fo- premaxillary foramen. Scale bar equals 5 cm.

The maxilla forms the ventral and anterior margins of the antorbital fenestra. In lateral view the anterior end tapers to a rugose point forming the premaxillary process of the maxilla. Just posterior to the premaxillary process a short anterolateral process inserts into the premaxilla in SMU 70635 and SMU 74749 (Fig 7). The process is more prominent on the larger specimen SMU 70635. This process is also present in *Haya griva* [15], *Orodromeus makelai* [16], and *Zephyrosaurus schaffi* [17]; however, it is more pronounced in these taxa than in *Convolosaurus marri*. Dorsal to the antorbital fossa the maxilla ascends as a thin sheet, curving posteriorly to contact the lacrimal, forming the anterior margin of the antorbital fenestra. The posterior margin of the maxilla contacts the jugal forming a butt joint. A maxillary fenestra is lacking in *Convolosaurus marri*, distinguishing it from *Hypsilophodon foxii* [18] and *Haya griva* [15].

The lateroventral portion of the maxilla bears 8 tooth positions in SMU 74749 and 10 tooth positions in SMU 70635 and SMU 72316 (Fig 7). The difference in tooth positions can be attributed to ontogenetic stage as SMU 74749 is smaller and presumably younger than the other two specimens. A prominent anteroposterior ridge runs along the lateral surface of the maxilla. The maxillary teeth in the type specimen, SMU 72834, become increasing medially inset from anterior to posterior as this ridge become more prominent (Fig 6). Ventral to the ridge and above the tooth row are nutrient foramina, which are seen in all neornithischians.

#### Nasal

In SMU 72316 (Fig 7) and SMU 74087 partial nasals are preserved contacting the premaxilla and anterior portions of the maxilla. The nasals are thin bones that are roughly triangular, domed, and have a slight midline depression as in most basal ornithopods. The anterior portion of the nasals form the dorsal and posterior corner of the narial opening. The dorsal processes of the premaxillae divide the anterior portion of the nasals and slightly overlap the medial portion of the nasals which is the condition present in *Hypsilophodon foxii* [18], but absent in *Haya griva* [15] and *Jeholosaurus shangyuanensis* [19]. The nasals come into medial contact posterior to separation by the dorsal premaxillary processes at one third to one half of their length. The nasals broaden posteriorly then taper to rounded or slightly pointed ends that lap onto the frontals. A slight notch separates the posterior ends of the nasals. The anterior frontals thin dramatically toward their contact with the nasals, well anterior to the posterior notch for the prefrontals. The posterolateral surface of the nasal is overlapped by the prefrontal. Foramina in the nasals are lacking as in *Hypsilophodon foxii* [18] and *Parksosaurus warreni* [20] whereas foramina are present in *Haya griva* [15], *Jeholosaurus shangyuanensis* [19], and *Thescelosaurus neglectus* [21].

#### Lacrimal

**Fig 8:**
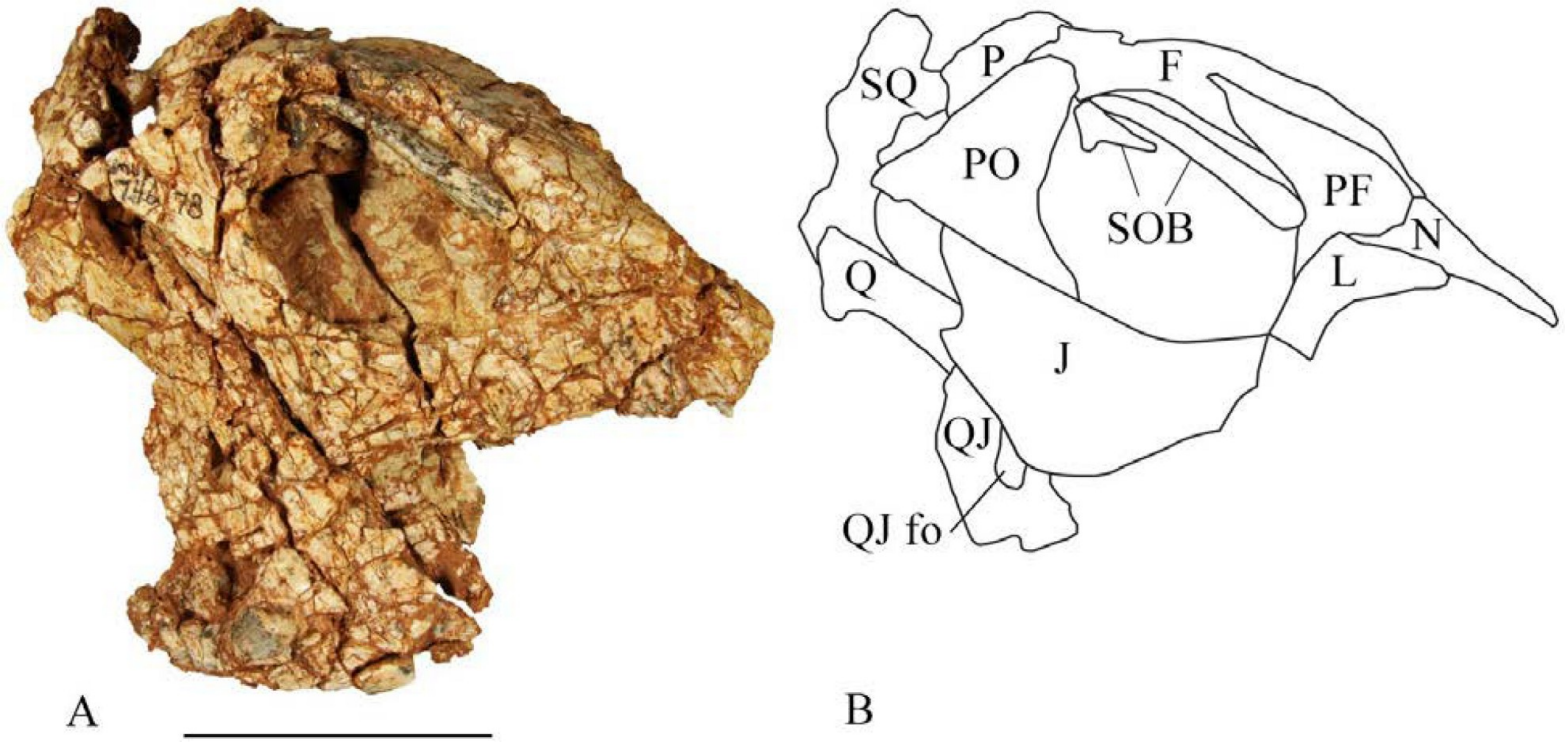
SMU 74678 cranium. (A) SMU 74678, posterior skull in right lateral view. This specimen contains two supraorbitals which are not fused to the frontal. (B) Outline drawing of right side of SMU 74678 posterior skull. Abbreviations: F-frontal, J-jugal, L-lacrimal, N-nasal, P-parietal, PF-prefrontal, PO-postorbital, Q-quadrate, QJ- quadratojugal, QJ fo- quadratojugal foramen, SOB-supraorbital, SQ-squamosal. Scale bar equals 5 cm.

Two partial lacrimals are represented in the left lateral view of SMU 74749 and right lateral view of SMU 74678 (Fig 8). The lacrimal forms the posterodorsal and dorsal margins of the antorbital fenestra, excluding the premaxilla from the antorbital fenestra. The posterior edge of the lacrimal forms part of the anterior margin of the orbit. The lateral surface is convex and is dorsal to both the jugal and maxilla similar to the condition in *Hypsilophodon foxii* [18]. The tip of the posteroventral process of the lacrimal barely contacts the anterior tip of the jugal. The posterodorsal corner of the lacrimal fits into the anteroventral corner of the prefrontal. The dorsal surface of the lacrimal contacts the ventral surface of the nasals.

#### Jugal

The jugals are poorly preserved in four specimens, mostly preserving only the anterior portion. The concave dorsal margin of the jugal forms the ventral margin of the orbit. It does not possess a pronounced jugal boss or ornamentation; however, SMU 74749 has several small foramina concentrated along lateral portions of the jugal (Fig 7). The maxilla and lacrimal exclude the anterior process of the jugal from the antorbital fenestra in SMU 74749 as is seen in *Hypsilophodon foxii* [18]. The anterior process of the jugal inserts into the maxilla, but the anterodorsal tip of the jugal barely contacts the posteroventral corner of the lacrimal. Medially the surface of the jugal is smooth. The posterior end rises dorsally and the posterodorsal end contacts the posteroventral edge of the postorbital forming an elongate scarf joint. The jugal flares dorsoventrally toward the posterior, overlapping the anterior lateral surface of the quadratojugal.

#### Quadratojugal

SMU 71631 and SMU 74678 (Fig 8) represent the most pristine quadratojugals of *Convolosaurus marri*. SMU 71631 is not articulated within a skull limiting its descriptive value, but it does appear to maintain its original contact with a partial jugal. SMU 74678 is articulated within a partial skull. The quadratojugal is subtriangular in lateral view and plate-like. The posterior end of the jugal significantly overlaps the anterior margin of the quadratojugal. A foramen pierces the center of the quadratojugal. Little can be determined from this specimen about the contribution of the quadratojugal to the infratemporal fenestra as the quadrate appears to have shifted. The contact between the quadratojugal and quadrate is preserved in SMU 74678 and quadrates preserved in SMU 75379 and SMU 74087 have concave anterolateral surfaces, which indicate the quadratojugal overlapped a significant portion of the quadrate shaft. A similar contact is seen in *Jeholosaurus shangyuanensis* [19], *Orodromeus makelai* [16], *Changchunsaurus parvus* [22], and *Parksosaurus warreni* [20], but differs from *Hypsilophodon foxii* [18] in which it does not extensively overlap the quadrate shaft.

#### Quadrate

SMU 75379 and SMU 74678 contain complete quadrates; however, they are semi-articulated and some contact surfaces are obscured. The quadrate consists of a vertical columnar shaft that bows anteriorly and has two thin sheets of bone extending anteriorly and anteromedially. The anterolaterally extending sheet is the moderately developed jugal wing, which begins a short distance below the dorsal head of the quadrate and extends to its ventral end. The quadratojugal significantly overlaps the lower half of the quadrate. The pterygoid wing extends anteromedially beginning from the dorsal head of the quadrate. The sheet expands medially but then begins to taper ventrally ending well above the distal condyles of the quadrate. The pterygoid wing is extensively overlapped by the pterygoid in SMU 72834. The lower third of the quadrate shaft is mediolaterally compressed and ends with equal distal condyles. The upper portion of the quadrate shaft is mediolaterally compressed with the head of the quadrate extending posterodorsally between the prequadratic and postquadratic processes of the squamosal.

#### Squamosal

**Fig 9:**
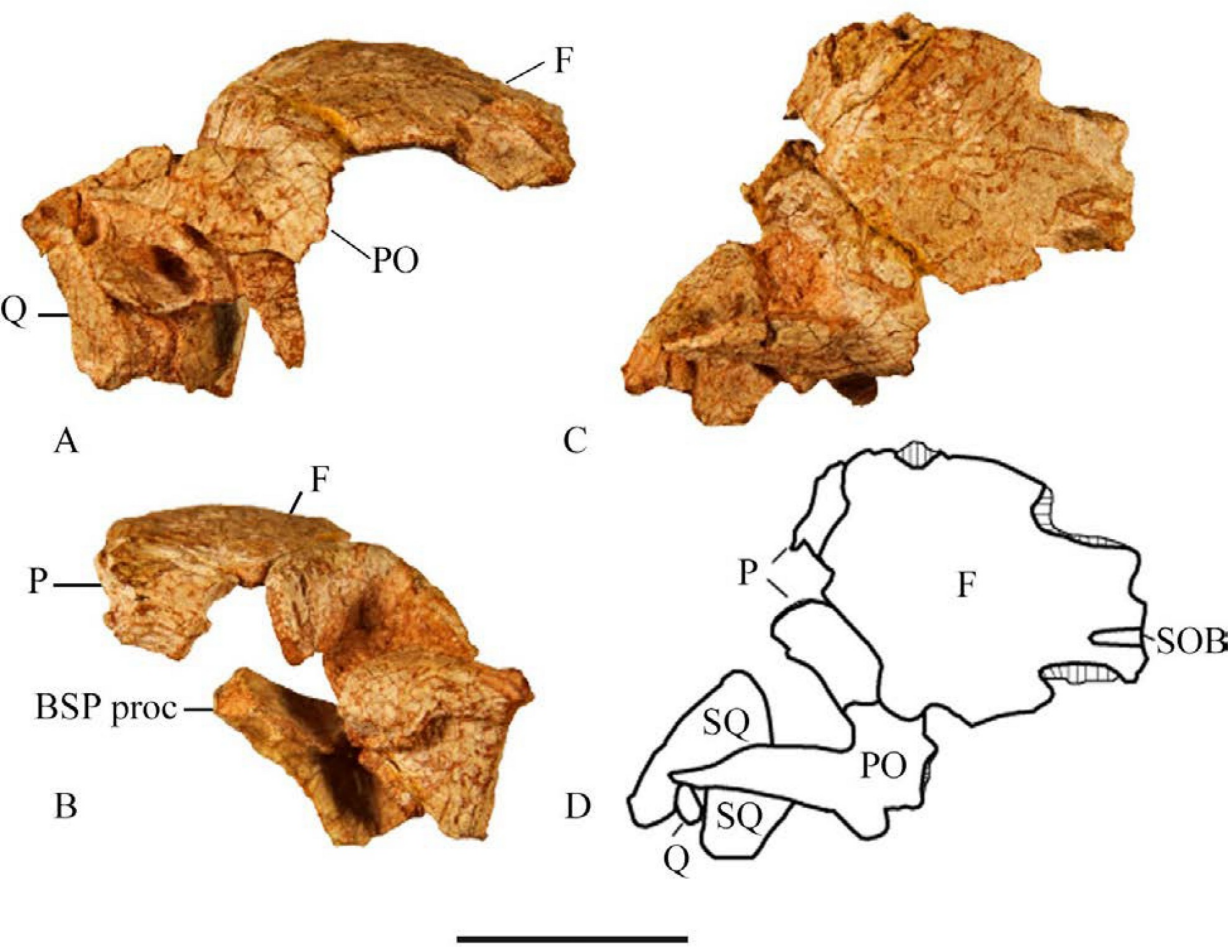
SMU 72834 partial cranium. (A) SMU 72834, posterior cranium in right lateral view. (B) SMU 72834, posterior cranium in posterior view. (C) SMU 72834, posterior cranium in dorsal view. (D) Outline drawing of dorsal view of SMU 72834. Abbreviations: BSP proc- basipterygoid process, F-frontal, P-parietal, PO-postorbital, SOB-supraorbital, SQ-squamosal, Q-quadrate. Scale bar equals 5 cm.

A partial right squamosal is preserved in the type specimen SMU 72834 (Fig 9) and in SMU 74678 (Fig 8). The main body of the squamosal forms the posterolateral margin of the supratemporal fenestra. Four processes then extend from the main portion, although only three are complete enough to describe. The postorbital process extends anteriorly; however, the anterior end is incomplete. Its dorsal surface contacts the ventral surface of the postorbital forming the dorsal margin of the infratemporal fenestra. Two processes extend from the dorsolateral corner of the main body: the prequadratic and postquadratic processes. In posterior view the postquadratic process forms a subtriangular tab that extends towards the lateral surface of the skull. It then contacts the posterior end of the quadrate head. The prequadratic process contacts the anterodorsal margin of the quadrate and extends anteroventrally.

#### Prefrontal

The prefrontal is preserved in SMU 74678 (Fig 8), forming the anterodorsal corner of the orbit. Its posteroventral surface articulates with a well-defined fossa on the frontal and its anterior portion overlaps the nasal. The anteroventral corner contacts the lacrimal. Its dorsal surface is slightly convex and the lateral surface is slightly concave. The orbital margin contains suture ridges forming an articulation surface for the supraorbital.

#### Frontal

The frontals are anteroposteriorly longer than they are wide and form the majority of the dorsal margin of the orbits as seen in basal ornithopods *Hypsilophodon foxii* [18] and *Parksosaurus warreni* [20]. The frontals are arched over the orbit and the orbital margins are rugose as is noted in *Haya griva* [15] and *Zephyrosaurus schaffi* [17]. The anterolateral surfaces of SMU 72834 (Fig 9), SMU 70456, and SMU 74087 contain a deep notch where the frontal articulates with the prefrontal. The posterolateral corner contains a sulcus that extends ventrolaterally where it articulates with the postorbital in a series of pronounced interlocking projections.

The posterior end of the frontal curves slightly downward and articulates with the parietals, forming a triple junction between the frontal, postorbital, and parietal. The suture between the parietals and frontals is straight with the posterior end of the frontals projecting a slight process into the parietals. The ventral surface contains a concave hour glass shape depression, which becomes wider and deeper in the posterior end and housed the olfactory bulb and tract and the anterior portion of the cerebrum. This is outlined by ridges created by the ventrolateral concavities of the orbits. The maximum thickness of the frontals varies amongst specimens. Measured at the posterior limit of the orbit, the thickness of the frontals ranges from 3.5 mm (maximum width across the frontals 22.3 mm) to 17.3 mm (maximum width across the frontals 59.7 mm) with an average of 10.7 mm amongst 10 specimens. These represent different ontogenetic stages; however, some variability could be a result of sexual dimorphism or individual variation.

#### Supraorbital

Four specimens contain partial supraorbitals; however, SMU 74678 (Fig 8) is the only specimen that preserves two supraorbitals or palpebrals, the second being much shorter and located at the posterodorsal corner of the orbit. The supraorbitals are free of the orbital margin and project across a significant portion of the orbit, as seen in *Agilisaurus louderbacki* [23] and *Thescelosaurus neglectus* [21]. It is a slender rod that is slightly wider than it is tall with a sharp lateral edge. The anterior end expands mediolaterally and its anteromedial surface articulates with the prefrontal. The supraorbital then tapers posteriorly ending in a subtriangular point at the posterior end of the orbit.

The accessory supraorbital is approximately half the length of the primary supraorbital. It is proportionally larger than the accessory supraorbital seen in *Agilisaurus louderbacki* [23], but is similar in proportion to the condition in *Thescelosaurus neglectus* [21]. It tapers anteriorly along its entire length, ending in a rounded point. The dorsal surface is slightly concave towards the anterior end, possibly serving as an articulation surface for the primary supraorbital. The ventral surface is slightly concave. Its lateral surface is a sharp edge similar to the primary supraorbital whereas the medial surface is fairly flat. The accessory supraorbital is disarticulated in SMU 74678, thus the exact articulation is unknown. Its anterior end likely contacted the posterior end of the primary supraorbital and then articulated with the posterior margin of postorbital as observed in *Thescelosaurus neglectus*.

#### Parietal

**Fig 10:**
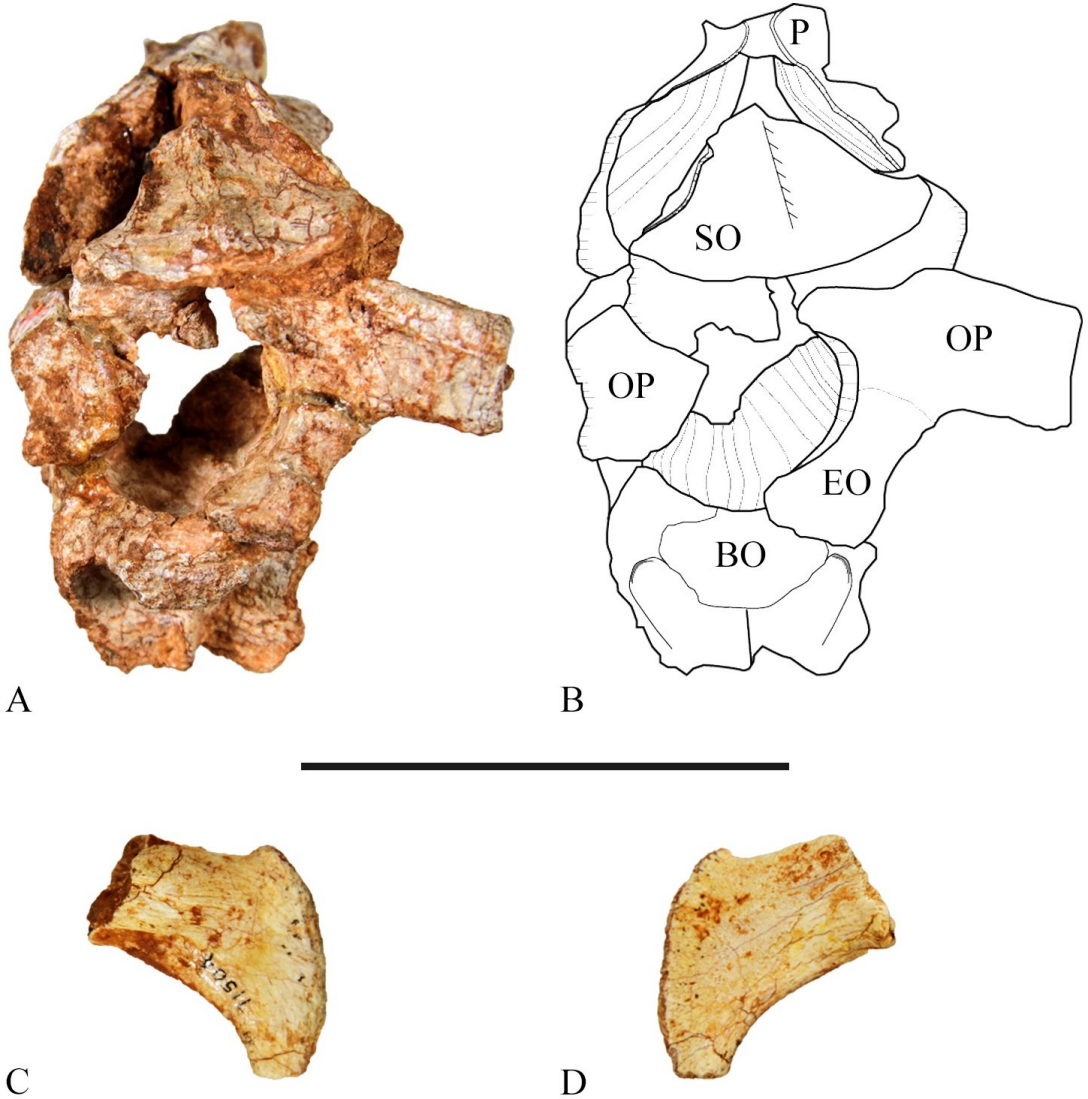
Occipital region of SMU 72834. (A) SMU 72834, occipital region in ventral view. (B) Outline drawing of SMU 72834, occipital region in ventral view. (C) SMU 71504, right distal paroccipital process in ventral view. (D) SMU 71504, right distal paroccipital process in dorsal view. Abbreviations: BO-basioccipital, EO-exoccipital, OP-opisthotic, P-parietal, SO-supraoccipital. Scale bar equals 5 cm.

Partial parietals are preserved in the type specimen (SMU 72834, (Figs 9 and 10) and in SMU 70456. The type specimen contains portions of the parietals which reveal lateral surfaces that are anteroposteriorly concave and transversely thin. In dorsal view the parietals slightly overlap the frontals except for the medial process of the parietal which is slightly overlapped by the frontals. A ridge is present on each parietal beginning at about the midpoint of the anterior edge. These ridges curve and converge posteriorly at the midline, following the lateral outline of the parietals, to form a weak crest. The anterolateral corners bear strong sutural ridges where they articulate with the frontals and postorbitals. The parietals are completely fused and posteriorly enclose the dorsal surface of the supraoccipital.

#### Postorbital

The anterior of the postorbital forms the posterodorsal corner of the orbit as it articulates with the posterolateral corner of the frontal (Fig 9). The orbital margin is smooth unlike the orbital margin formed by the frontals. The anterodorsal corner of the postorbital articulates to the frontal and parietal forming a triple junction consisting of pronounced interlocking projections as in *Hypsilophodon foxii* [18], *Zephyrosaurus schaffi* [17], and *Orodromeus makelai* [16]. The postorbital splits into two distinct processes directed ventrally and posteriorly that occur in the same plane. The ventral process tapers to a point and is anteriorly concave with the anterior edge defining the posterior rim of the orbit. The posterior edge of the ventral process forms part of the anterodorsal margin of the infratemporal fenestra. This ventral process contacts the posterodorsal surface of the jugal. The relatively thin posterior process of the postorbital also tapers to a point and with a concave posterior surface which forms the anterolateral margin of the supratemporal fenestra. This is overlapped onto the squamosal, but the contact is not well preserved in the specimens being described.

The medial surface of the postorbital has a continuation of the ridge from the frontals and a ridge that continues from the parietals. These two ridges run dorsoventrally and meet at the ventral process forming a more prominent ridge. This creates a synovial socket just ventral to the junction of the ridges where the head of the laterosphenoid articulated. This socket extends onto parts of the parietal and frontal as it occurs at the triple junction of these bones as is common for small ornithopods.

#### Pterygoid

The type specimen (SMU 72834) contains the dorsal portion of the right pterygoid. It comprises a thin sheet that overlaps the pterygoid process of the quadrate and angles medially. The medial margin is concave and articulates with the posterolateral margin of the basipterygoid process of the basisphenoid.

#### Supraoccipital

The base of the supraoccipital forms part of the dorsal boundary of the foramen magnum, but it is restricted by the medial process of the exoccipital. The posterior surface is triangular and narrows dorsally as it is wedged between the parietal wings. In SMU 72834, the anterodorsal end of the supraoccipital is straight rather than pointed (Fig 10). The posterior surface contains a median ridge that runs the entire length of the surface as seen in other basal ornithischians such as *Hypsilophodon foxii* [18]. The supraoccipital sweeps anteriorly under the parietal wings where it likely contacts the laterosphenoid; however, this contact is not present in SMU 72834 as the laterosphenoid is missing. The opisthotic is sutured to the posterolateral corner of the supraoccipital with a wide square contact. The prootic is sutured along the arched ventral edge of the supraoccipital. This sutural junction is excavated medially to form the fossa subarcuata which housed the floccular lobes of the cerebellum [18].

#### Exoccipital and opisthotic

The exoccipital forms the ventrolateral border of the foramen magnum and its medial surface forms part of the occipital condyle. Its ventral surface is then strongly sutured to the basioccipital. The opisthotic forms the lateral wall of the foramen magnum. Most of the anterior surface of the opisthotic is sutured to the supraoccipital and its median anterior corner is sutured to the prootic. The fenestra ovalis, middle ear cavity, internal auditory meatus, and the jugular foramen are situated between the junction with the opisthotic, prootic, and basioccipital. The lateral extent and paroccipital process of the opisthotic fused with the exoccipital is incomplete in SMU 72834. SMU 71695 and SMU 71504 contain distal portions of the paroccipital process revealing a laterally compressed bone that curves ventrally and is pendent shaped (Fig 10).

#### Basioccipital

The dorsal surface of the basioccipital forms the posteroventral floor of the braincase. The exoccipital and opisthotic are sutured to its dorsal surface at the posterolateral corners. The basioccipital forms most of the occipital condyle with a bulbous smooth articular surface posteriorly. A ventral keel extends along the ventral midline, which branches in the posterior half to create a large foramen that penetrates dorsally into the basioccipital; however, it does not appear to penetrate the floor of the braincase. In ventral view the anterior end of the basioccipital forms a ‘V-shape’ which creates an anterolateral contact surface with the basisphenoid. This distinguishes it from *Hypsilophodon foxii* [18] whose basioccipital and basisphenoid are fused.

#### Prootic

The type specimen (SMU 72834) contains a partial right prootic, which forms part of the lateral wall of the braincase. The dorsal surface is dorsoventrally convex and articulates posteriorly with the supraoccipital. The posterior end of the prootic tapers to a subtriangular process and is sutured to the opisthotic. Ventrally it is sutured to the basioccipital posteriorly and to the basisphenoid anteriorly. The fenestra ovalis is situated between the junction of the prootic, opisthotic, and basioccipital. Dorsomedially near the suture with the supraoccipital the shallow fossa subarcuata is preserved. The contact between the prootic and laterosphenoid is not preserved in the available specimens.

#### Basisphenoid

Only the type specimen (SMU 72834) contains portions of the basisphenoid. This includes two small pieces revealing the posterodorsal region of the basisphenoid, which articulates with the posterior surface of the prootic and the anterior margin of the basioccipital. These two pieces are concave in lateral view, but little else can be described. A separate piece contains portions of the right basipterygoid process of the basisphenoid, which articulates with the pterygoid. The piece is triangular in cross section and its dorsal surface is concave. The anterior surface is slightly convex and the posteroventral surface reveals two bifurcating ridges that form a subtriangular depression.

#### Predentary

A well preserved predentary is articulated in SMU 70444 and is figured in Winkler et al., 1988. The length of the predentary is approximately the same length as the premaxilla. The oral margin is sharp. The predentary has posterolateral processes and a posteroventral process. Together they form the articulation surface for the dentary creating a u-shaped sulcus. The posterolateral process extends laterally fitting into the anterodorsal margin of the dentary. The posteroventral process connects to the ventral surface of the dentaries and extends farther posteriorly than the posterolateral processes. The single posteroventral process is not bifurcated. The lateral surface of the predentary contains one prominent foramen and four smaller foramina which are concentrated toward the anterior margin. A shallow sulcus extends posteriorly beginning near the anterior tip and runs along the lateral surface through the lateral foramen. This sulcus ends near the posterolateral process. A similar sulcus is present in *Hypsilophodon foxii* [18] and *Thescelosaurus neglectus* [21], but in *Convolosaurus marri* it is not as prominent as the sulcus present in *Changchunsaurus parvus* [22] and *Jeholosaurus shangyuanensis* [19]. In ventral view the anterior end of the predentary is rounded distinguishing it from neornithischians *Jeholosaurus shangyuanensis* [19], *Thescelosaurus neglectus* [21], *Changchunsaurus parvus* [22], and *Hypsilophodon foxii* [18] who all display sharply pointed anterior ends.

**Dentary**: The right dentary on the type specimen (SMU 72834) is 125 mm and contains seven teeth (Fig 6), but based on observed alveoli and spacing could accommodate up to 11 teeth. The tooth row of SMU 70444 is 39 mm and contains eight dentary teeth, whereas the dentary of SMU 74087 is 52 mm and contains 11 tooth positions, indicating ontogenetic variation in tooth count. A lateral ridge, approximately 2 cm below the dorsal margin, begins at the anterior end of the dentary and becomes more pronounced as it continues posteriorly. Alveoli occur dorsal to the lateral ridge and become moderately inset posteriorly as the lateral ridge becomes more prominent. The most anterior alveoli are smaller and begin directly posterior to the contact with the predentary. A row of nutrient foramina lies ventral to the tooth row on the lateral surface of the dentary.

The dorsal and ventral margins of the dentary are parallel posteriorly, but the anterior portion of the dentaries turn medially, converging to form a spout-shaped symphysis. The anterior end of the dentary tapers into a rugose process with three foramina that inserts into the predentary. Beginning anteriorly at the predentary articular surface, the Meckelian groove runs along the ventromedial margin of the dentary. The groove widens and deepens as it continues posteriorly to become covered by the splenial. The posterodorsal margin of the dentary forms the anterior portion of the coronoid process (Figs 6 and 7). This process rises dorsally and curves posteriorly. The posterior end of the tooth row in SMU 70444 and SMU 72834 extends one tooth medial to the coronoid process. The posterior margin of the dentary contacts the surangular and angular while the coronoid contacts the dentary medially behind the coronoid process.

#### Splenial

The splenial is visible in the type specimen SMU 72834 and forms the anteromedial section of the mandible. The ventral margin is anteroposteriorly convex. The splenial tapers both anteriorly and posteriorly forming subtriangular shaped ends. The splenial is medial to the angular so that its lateral surface contacts the medial surface of the angular.

#### Angular

The angular is a thin sheet that in lateral view comprises the posteroventral portion of the mandible. The ventral margin is anteroposteriorly convex. The anterior limit is positioned medial to the dentary and is overlapped medially by the splenial. The contact with the articular and prearticular are obscured.

**Surangular**: None of the specimens contain a complete surangular but several key features are preserved. The anterior margin of the surangular contacts the dentary. The ventral margin contacts the angular. A foramen measuring 3 mm across is present on the lateral surface at the contact between the surangular and dentary just below the coronoid process in specimen SMU 70444. The posteroventral portion of the surangular tapers ventrally, but upturns dorsally at its termination to form the retroarticular process. This process bears a foramen on the posterolateral surface in SMU 75379 and SMU 71510.

### Dentition

#### Premaxillary dentition

Four premaxillary teeth are preserved in each premaxilla of SMU 70444, SMU 74087, SMU 72316 (Fig 5), and SMU 75380. The anteroposterior tooth row length of the smallest of these premaxilla, SMU 75380, measures 11 mm, and the largest, SMU 72316, measures 25 mm. The specimens are fragmentary, but the tooth count is clearly four despite their varying size and presumably ontogenetic stage. The presence of four premaxillary teeth distinguishes this taxon from other basal ornithischians which typically contain five premaxillary teeth. The premaxillary teeth are equally sized in each individual with larger individuals displaying proportionally larger premaxillary teeth. The crown of the premaxillary teeth are buccolingually compressed and the pointed crown recurves posteriorly. The anterior and posterior edges of the crown bear fine serrations similar to *Hypsilophodon foxii* [18]. The serrations extend farther toward the base of the crown on the posterior edge than the anterior. The serrations are weaker or absent on the anterior edge likely related to higher degree of wear. Buccal and lingual surfaces of the crown are evenly enameled and ornamented with six to eight fine ridges similar to *Hypsilophodon foxii* [18] and *Thescelosaurus neglectus* [21]. The buccal surface of the crown possesses a shallow sulcus that runs proximodistally, separating the tooth surface into two lobes (Fig 5). This feature is unique to *Convolosaurus marri*. The bases of the premaxillary teeth are slightly constricted and the roots are circular in cross section.

#### Maxillary dentition

**Fig 11:**
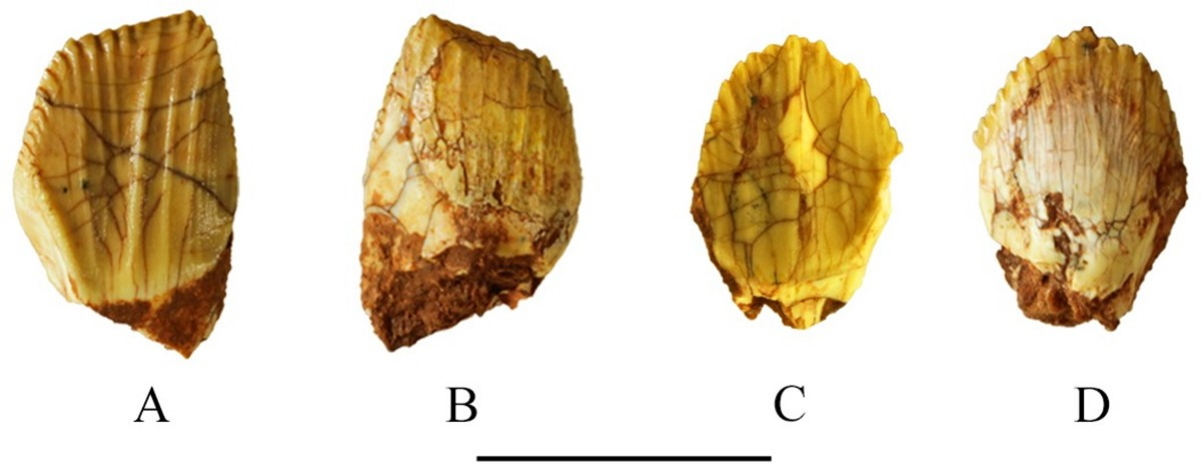
SMU 72316 Maxillary and dentary teeth. (A) SMU 72316, maxillary tooth lateral view. (B) SMU 72316, maxillary tooth medial view. (C) SMU 72316, dentary tooth medial view. (D). SMU 72316, dentary tooth lateral view. Scale bar equals 1 cm.

The tooth row length of SMU 74749 is 38 mm and contains eight maxillary tooth positions. The tooth row lengths of type specimen (SMU 72834), SMU 70635, SMU 72316 measure 87 mm, 59 mm, and 60 mm, respectively, and each bears ten maxillary tooth positions which are inset medially. The tooth count clearly varies with ontogenetic stage as SMU 74749 is significantly smaller compared to the other two specimens. The maxillary teeth are slightly obliquely aligned with the anterior tooth slightly overlapping onto the buccal surface of its posterior neighbor. The maxillary crowns are laterally compressed, forming a lozenge-like shape with denticulate margins (Fig 11). The apex of the maxillary crown is distinctly asymmetric in buccal view with the apex being offset posteriorly. Enamel is primarily restricted to the buccal side of the maxillary crown. Numerous ridges are present on the enameled buccal surface which are confluent with the denticles and extend to the base of the crown. These ridges are of equal prominence. Margins of the maxillary crowns contain up to 17 denticles, but have only six to seven ridges, thus the number of ridges varies and not all denticles are supported by ridges. The maxillary crown tapers to the root where its base swells slightly forming a cingulum. Maxillary tooth roots are curved medially in anteroposterior view distinguishing *Convolosaurus marri* from *Hypsilophodon foxii* [18].

#### Dentary dentition

Five specimens contain *in situ* dentary teeth; however, the tooth count varies based on preservation and ontogenetic stage, thus the exact number and variation of the dentary teeth within the dentary is unknown. The type specimen SMU 72834 contains seven teeth, but based on spacing it could have held up to 11. The right dentary of SMU 74087 measures 52.2 mm in length and contains *in situ* teeth, tooth roots, and alveoli suggesting 11 teeth were present. A smaller and presumably younger individual, SMU 70444, contain eight teeth with the tooth row measuring 39 mm, again showing variance in tooth count due to ontogenetic stage. The dentary crowns are laterally compressed with a spade-like shape, denticulate margins, and a cingulum at the base of the crown (Fig 11). However, in dentary teeth the enamel and ridges are restricted to the lingual side of the tooth, which contains a prominent apical ridge that runs down the center of the crown toward the base. Six or seven secondary ridges extend from the margins of the crown to its base; however, as in the maxillary teeth there are fewer ridges than denticles present on the crown, similar to *Hypsilophodon foxii* [18]. Margins of the dentary crown contain up to 14 denticles. The dentary crowns are symmetrical with the apex occurring at the center of the crown. The roots of the dentary teeth are also curved medially in anteroposterior view.

### Postcranial skeleton

#### Proatlas and atlas

The type specimen (SMU 72384) preserves portions of the proatlas and atlas; however, they are obscured from view and cannot be examined as further mechanical preparation would lead to destruction of the type skull.

#### Axis

**Fig 12:**
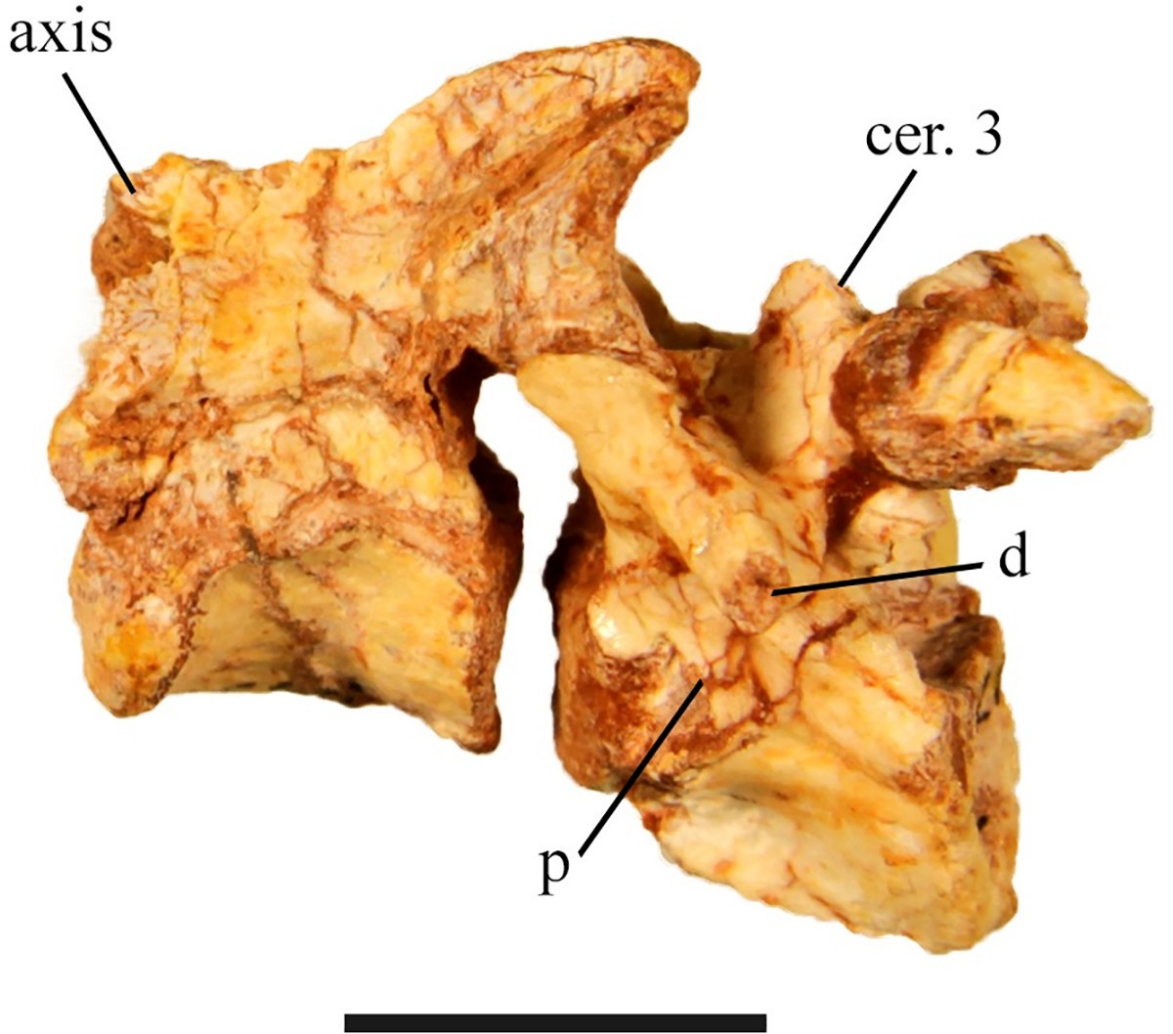
SMU 75621 axis and third cervical vertebra. Axis and third cervical vertebrae in left lateral view. Abbreviations: d-diapophysis, p-parapophysis. Scale bar equals 1 cm.

SMU 75621 (Fig 12) and the type specimen (SMU 72834) reveal an opisthocoelus centrum that is longer than it is wide and is ventrolaterally concave with a moderate keel on its ventral surface. The neural arch is compressed dorsally forming a well-developed neural spine. The neural spine extends posterodorsally over the third cervical vertebra to the prezygapophyses. This extension contains well-developed postzygapophyses, which articulate with the prezygapophyses of the third cervical vertebra. The prezygapophyses of the axis are less developed, but articulate with the atlas. The diapophysis is small and occurs along the suture between the centrum and neural arch. The parapophysis is absent or not preserved in the available specimens.

#### Cervical vertebrae

**Fig 13:**
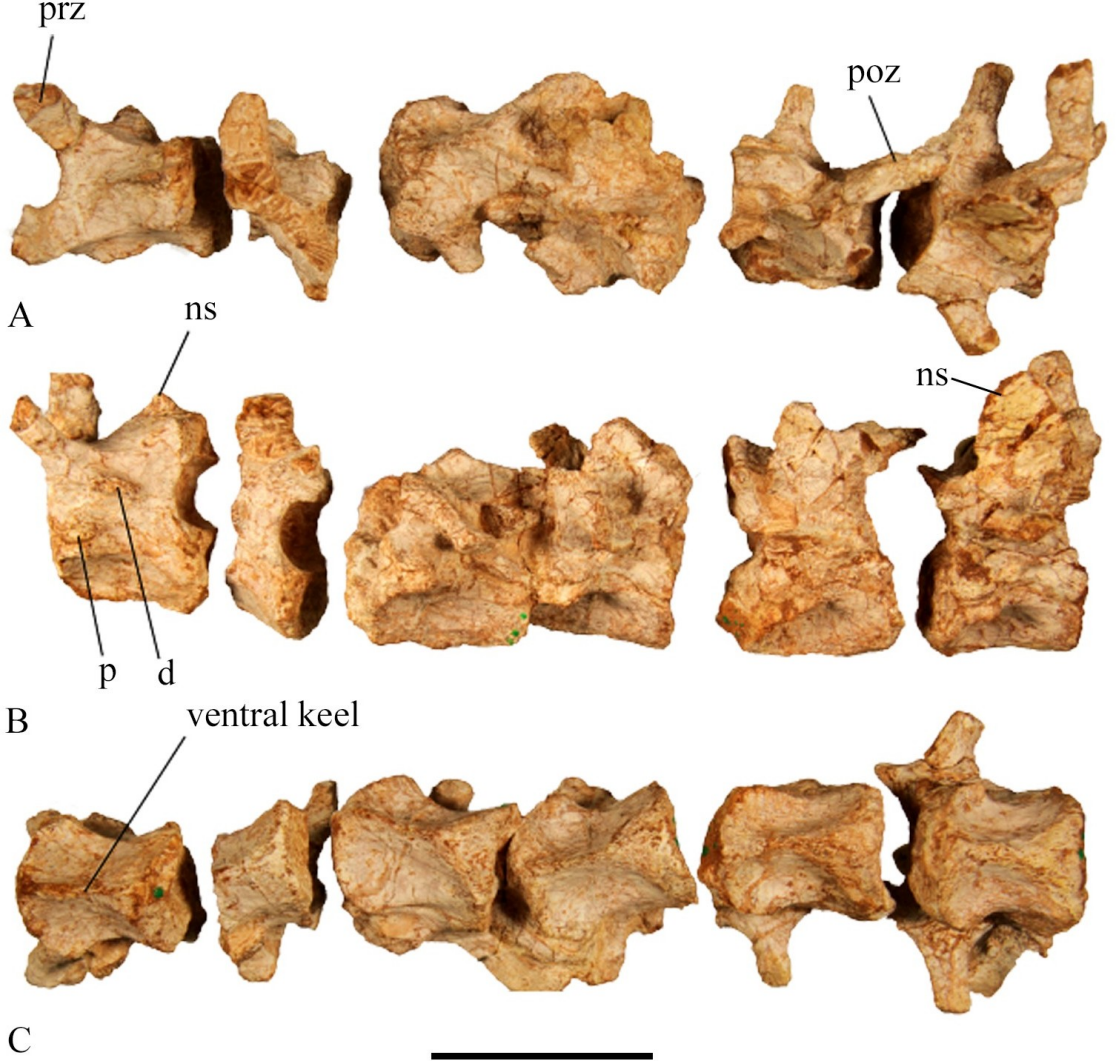
SMU 72834 cervical vertebrae. (A) Dorsal view of cervical vertebrae 4–9. (B) Left lateral view of cervical vertebrae 4–9. (C) Ventral view of cervical vertebrae 4–9. Abbreviations: d-diapophysis, ns-neural spine, p-parapophysis, prz-prezygapophyses, poz-postzygapophyses. Scale bar equals 5 cm.

The type specimen (SMU 72834) contains 9 cervical vertebrae (Fig 13). The cervical centra are opisthocoelus with an oval anterior face and D-shaped posterior face. The ventral surface of the centra are strongly concave in lateral view with a sharp ventral keel that broadens in progressively posterior cervical vertebrae. The neurocentral suture is visible in all the cervical vertebrae. It intersects the parapophysis on the anterolateral surface. Beginning with the axis, the transverse processes increase in length, carrying the diapophysis on its distal tip and migrate anterodorsally along the prezygapophyses.

The neural spine in post-axial cervical vertebrae rises on the posterior end of the neural arch slightly anterior of the postzygapophyses. The spines become progressively taller and more pronounced in succeeding vertebrae with the largest neural spine equaling the height of the centrum in cervical 9. The neural spines also migrate anteriorly in succeeding cervical vertebrae. The prezygapophyses on anterior cervicals are short paddle shaped extensions with a broad articular surface, but they become progressively smaller and more spike shaped along the cervical column. The postzygapophyses that can be observed are larger than the prezygapophyses. The zygapophyses arise higher on the neural arch and become larger posteriorly. The four cervical ribs preserved in SMU 74749 show progressively longer ribs proceeding posteriorly.

#### Dorsal vertebrae

**Fig 14:**
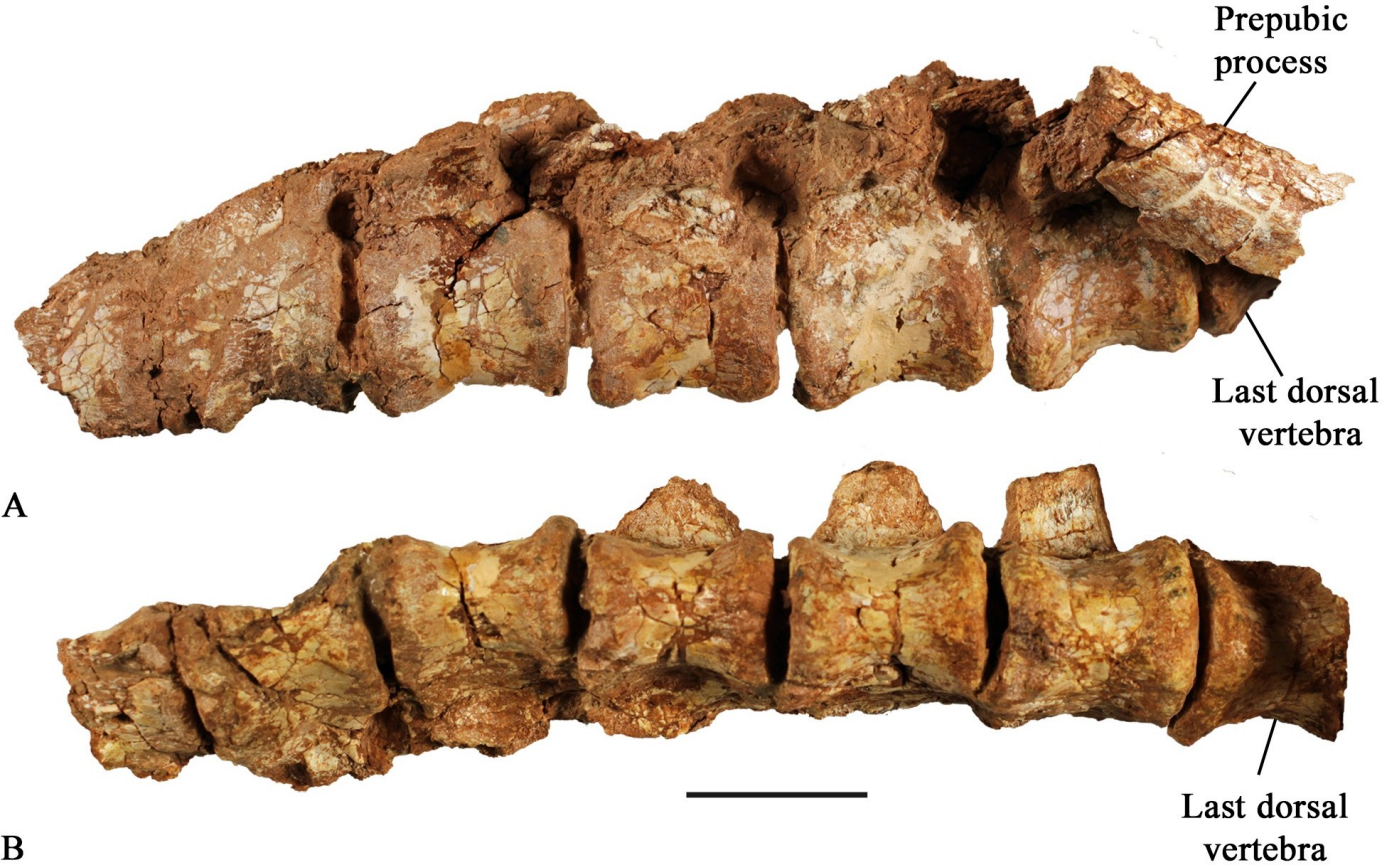
SMU 72316 dorsal vertebrae. (A) Left lateral view of dorsal vertebrae nine through 15. (B) Ventral view of dorsal vertebrae nine through 15. Scale bar equals 3 cm.

The type specimen (SMU 72834) preserves 15 dorsal vertebrae. SMU 72316 contains 8 articulated dorsal vertebrae representing the posterior dorsal vertebrae as they articulate to the sacrum (Fig 14). SMU 70456 contains 11 articulated dorsal vertebrae with associated ribs although several anterior vertebrae are missing. The ventral surface of the most anterior centra are sharply keeled with ventrolaterally concave surfaces. Posteriorly the keel diminishes becoming a smooth rounded surface. The centra become wider and more robust posteriorly (Table 1). The anterior faces of the first eight centra are smaller than the posterior faces and are taller than they are wide. The anterior faces of the centra become progressively wider in succeeding vertebrae until the last dorsal vertebra which has equally sized anterior and posterior faces. The dorsal centra are amphicoelous and contain muscle insertion scars along the edges of the anterior and posterior margins, especially along the ventral surface. The diapophysis and parapophysis become progressively closer together in succeeding vertebrae until they are united in the last two dorsal vertebrae. The prezygapophyses and postzygapophyses become slightly longer in succeeding vertebrae with an articular surface 45 degrees from the horizontal plane. The postzygapophyses extend slightly further than the prezygapophyses. The transverse processes arise lower on the neural arch in succeeding dorsal vertebrae. The transverse processes also shorten posteriorly until the parapophysis and diapophysis unite as one facet on the last two dorsal vertebrae. The neural spines become thicker and anteroposteriorly longer down the dorsal vertebral column. From anterior to posterior the dorsal ribs become progressively shorter.

**Table 1:** Skeletal measurements of *C*. *marri*, sp. nov. (in mm). Left and right are indicated along with estimated position of the vertebrae. Abbreviations: Ht-height; L-length; W-width; dist-distal; dor-dorsal; lt-left; pr-proximal; rt-right; vent-ventral.

Element	SMU 72834	SMU 72316	SMU 70534
Cervical 4- Centrum L ˣ W	41 ˣ 35	―	―
Cervical 7- Centrum L ˣ W	38 ˣ 33	―	―
Dorsal 10- Centrum L ˣ W	38 ˣ 36	31 ˣ 26	―
Sacrum- L	228	158	―
Caudal 1- Centrum L ˣ W	33 ˣ 35	27 ˣ 30	―
Caudal 10- Centrum L ˣ W	48 ˣ 37	41 ˣ 32	―
Caudal 20- Centrum L ˣ W	―	42 ˣ 18	―
Caudal 30- Centrum L ˣ W	―	36 ˣ 14	―
Scapula- L	239 (rt)	―	―
Coracoid- Ht. (dor./vent.) ˣ W (pr)	88 ˣ 71 (rt)	―	―
Humerus- L ˣ prox. W	221 ˣ 63 (lt)	―	―
Ulna- L	182 (lt)	―	―
Radius- L	169 (lt)	―	―
Femur- L ˣ prox. W ˣ dist. W	316* ˣ 70 ˣ 85 (rt)	282* ˣ ― ˣ 77 (lt)	―
Tibia- L ˣ prox. W ˣ dist. W	362* ˣ 94 ˣ ― (lt)	314* ˣ 80 ˣ ― (lt)	345 ˣ 68 ˣ 94 (lt)
Fibula- L	―	―	301 (lt)
Metatarsal I- L	―	―	72 (lt)
Metatarsal II- L	―	―	121
Metatarsal III- L	―	―	143
Metatarsal IV- L	―	―	112
Metatarsal V- L	―	―	52

*Estimated

#### Sacral vertebrae

**Fig 15:**
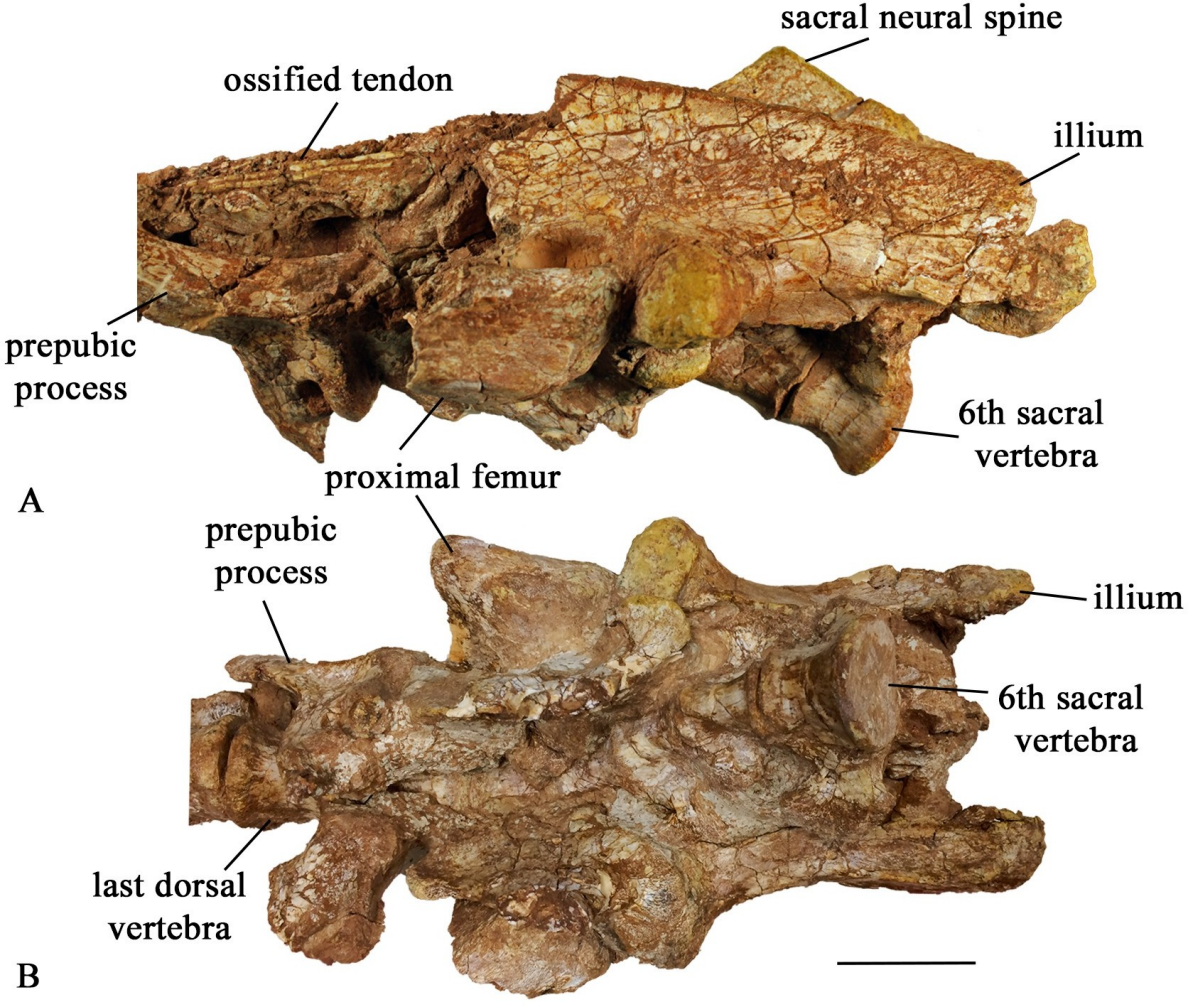
SMU 72316 pelvic girdle. (A) SMU 72316, pelvic girdle left lateral view. (B) SMU 72316, pelvic girdle ventral view. Scale bar equals 5 cm.

The type specimen (SMU 72834), SMU 72316, and SMU 70456 contain articulated sacra. SMU 72316 has the best-preserved sacrum, including 6 sacral vertebrae in articulation with dorsal and caudal vertebrae (Fig 15). The first two sacral centra are fused; however, the remaining four are separate. The posterior end of centrum 6 is slightly expanded and is concave receiving the anterior end of the first caudal vertebra. The ventral surface of the centrum is smooth and round. The last three sacral ribs are visible in SMU 72316 (Fig 15) with the third sacral rib contacting the medial surface of the ischial peduncle and sacral ribs four and five contacting the brevis shelf of the ilium. The sacral neural spines are greater than twice the height of the sacral centra distinguishing this taxon from *Hypsilophodon foxii* [18].

#### Caudal vertebrae

**Fig 16:**
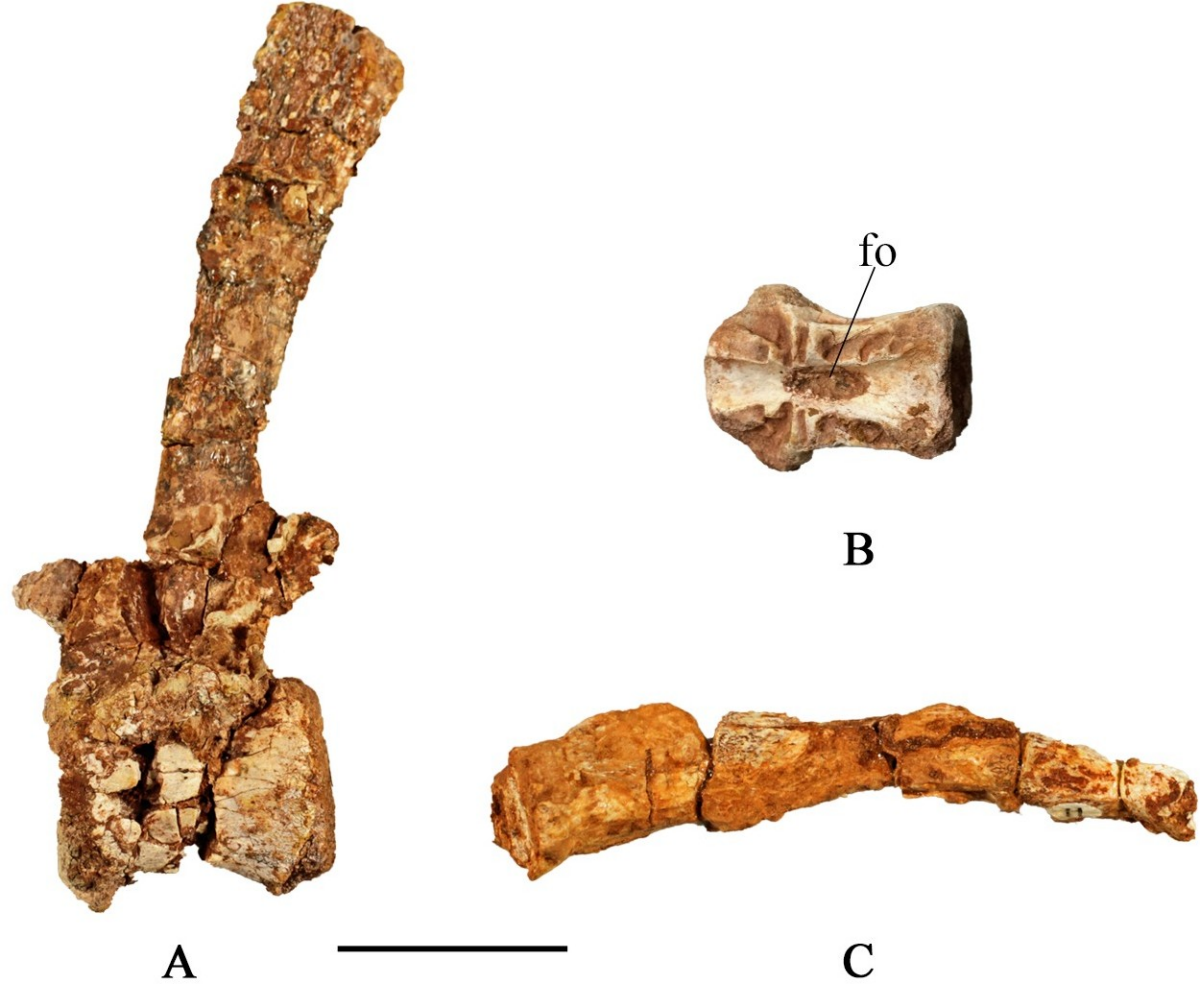
Caudal vertebrae. (A) SMU 70456, proximal caudal vertebra in left lateral view. (B) SMU 71492, caudal centra in dorsal view displaying anteroposteriorly elongate foramen. (C) SMU 72316, distal caudal vertebrae (#32–34) in left lateral view. Abbreviations: fo-foramen. Scale bar equals 3 cm.

A nearly complete caudal series is preserved in SMU 72316, containing 43 articulated vertebrae. The type specimen (SMU 72834) preserves a partial caudal series, containing 23 caudal vertebrae. Proceeding posteriorly the height and width of the centra decrease as their length increases (Fig 16; Table 1). This trend gradually occurs in caudal vertebrae 1–30 at which point the length of the vertebrae begins to decrease at the most distal portions of the caudal series. Anterior centra are ventrolaterally concave; however, this becomes less prominent in succeeding posterior centra, creating a transition from rounded ventral surfaces to more flat ventral surfaces by caudal vertebra 10. The ventral surface of the neural canal bears an anteroposteriorly elongate foramen in several disarticulated centra (Fig 16).

Caudal vertebra 10 is the first in the series without caudal ribs. The transverse processes attach at the neurocentral suture and are fused in SMU 72316. The length of the transverse processes of the anterior caudals are approximately equal to the height of the neural spine. The longest caudal rib is found on caudal vertebra five. The longest caudal ribs preserved are slightly longer than the width of the centra. The first caudal rib sweeps posteriorly, but proceeding ribs extend straight laterally. The prezygapophyses become progressively thinner in posterior vertebrae, but maintain approximately constant length.

The neural spines of the first nine caudal vertebrae are situated on the posterior half of the centra and slightly recline posteriorly or are nearly vertical. They slightly increase in height from caudals 1–9, but all of them are greater than 1.5 times the height of their respective proximal caudal centra. Posterior to caudal vertebra 9 the neural spines decrease in height and become progressively wider anteroposteriorly at their base. Caudal vertebra 29 contains the most posterior preserved neural spine although it seems likely that neural spines were present, but not preserved in more posterior vertebra. Although the caudal series is not complete in SMU 72316, *C*. *marri* probably did not contain an elongate tail with more than 59 caudal vertebrae given the size and morphology of the last preserved caudal vertebra in the series, distinguishing it from *Tenontosaurus* [13]. Articulated chevrons present in caudals 8–26 become progressively shorter. The chevrons articulate to the posteroventral surface of the associated caudal centra. The chevrons are round in cross section at midshaft, but become laterally compressed distally. As a result, the distal portion of the chevrons becomes expanded in lateral view and wider than the proximal articular surface.

#### Ossified tendons

**Fig 17:**
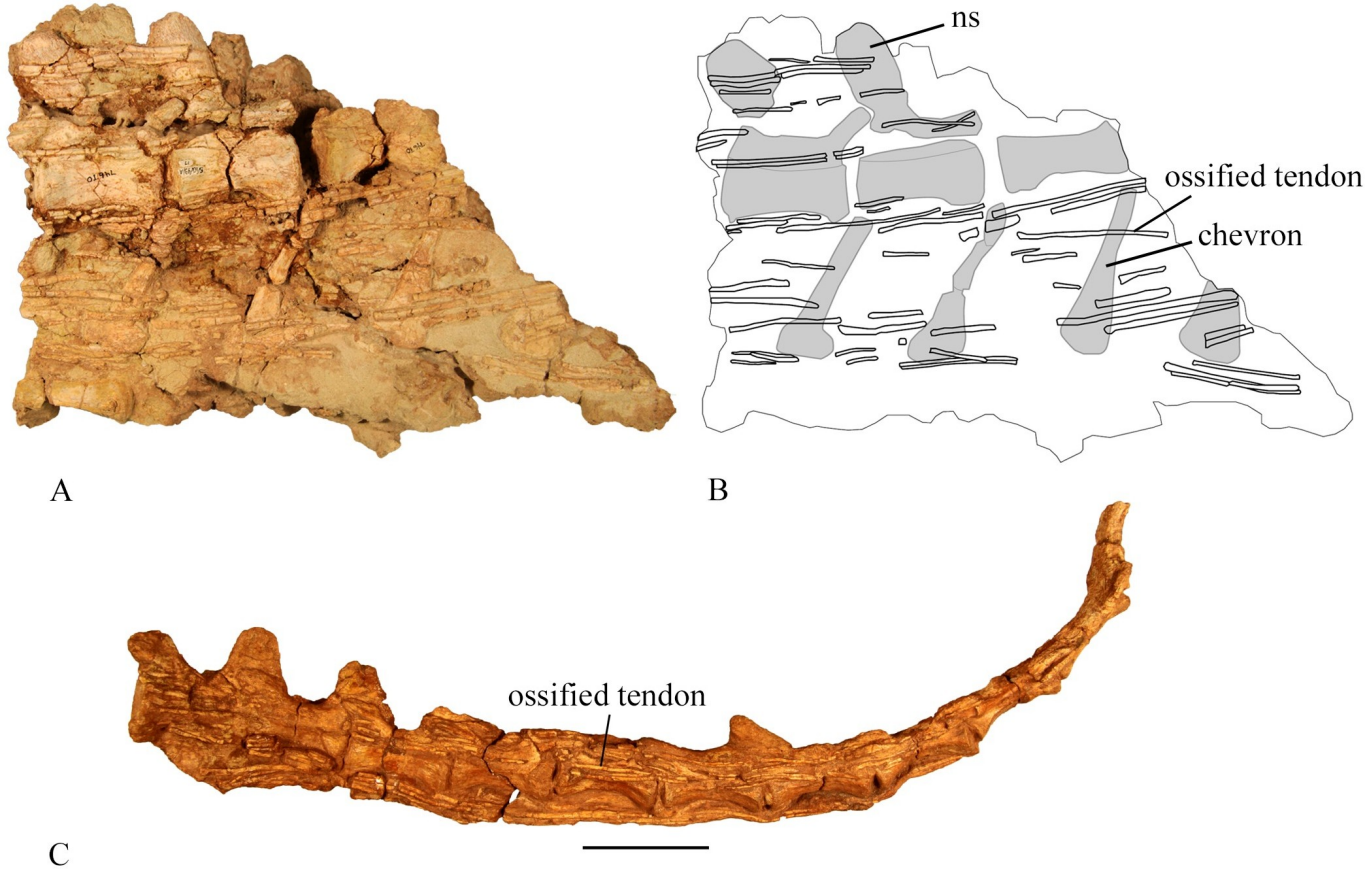
Ossified tendons in the caudal region. (A) SMU 74670, caudal vertebrae with ossified tendons preserved in right lateral view. (B) Outline drawing of SMU 74670, caudal vertebrae and ossified tendons. (C) SMU 72316, articulated caudal vertebrae (#16–26) with ossified tendons preserved in left lateral view. Abbreviations: ns-neural spine. Scale bar equals 5 cm.

Ossified tendons are preserved in SMU 70456, SMU 72316, SMU 74610, and SMU 74576 (Fig 17). This includes tendons along the dorsal, sacral and caudal sections of the vertebral column. Preserved tendons in the dorsal section are prominently displayed in SMU 70456. Individual tendons extend the length of two dorsal vertebrae. The tendons run parallel to each other and not in the rhomboidal lattice-like arrangement present in more derived iguanodonts [24]. As many as nine tendons are preserved parallel to one another on one side of a single dorsal vertebrae. Additional tendons may have been lost through preservation and possibly preparation. Tendons preserved in the sacral section have the same arrangement as those in the dorsal section although fewer tendons are visible and are slightly thicker.

SMU 72316 contains ossified tendons in the caudal region. Caudals 9–16 contain parallel epaxial and hypaxial tendons. Three to four parallel tendons occur in this section with individual tendons extending the length of 2 centra. Along caudals 16–43, as many as 8 tendons lie parallel. SMU 74670 contains 5 vertebrae in the range of caudals 10–15 based on vertebral size and morphology. This section preserves as many as 18 tendons running parallel along the length of a single centrum. Epaxial and hypaxial tendons extend from the dorsal surface of the neural spine to the ventral surface of the chevrons.

#### Sternal plates

No sternal plates have been recovered from the Proctor Lake locality. Due to the phylogenetic distribution of sternal plates that are found in neornithischian taxa the absence of sternal plates probably represents preservational bias rather than the absence of these bones from *Convolosaurus marri*.

#### Scapula

The scapula is slightly longer than the humerus in larger specimens including the type specimen (SMU 72834) where the scapula is 17 mm longer than the humerus; however, the scapula and humerus are approximately equal in smaller individuals including SMU 74664 where the humerus and scapula both measure 75 mm in length. The scapula is a short, broad, blade shaped element with a length approximately 6 times that of its minimum width. The blade strongly expands distally with a distal width two and half times that of the neck width. The interlocking projections on the articular surface form a tight suture with the coracoid; however, the scapula and coracoid are not fused in any of the specimens. The low, broad scapular spine overhangs the coracoid articulation, but is less pronounced than the sharp scapular spines found in specimens of *Orodromeus makelai* [16] and *Oryctodromeus cubicularis* [25]. The glenoid is broad and smooth and projects beyond the ventral margin of the scapular shaft.

**Fig 18:**
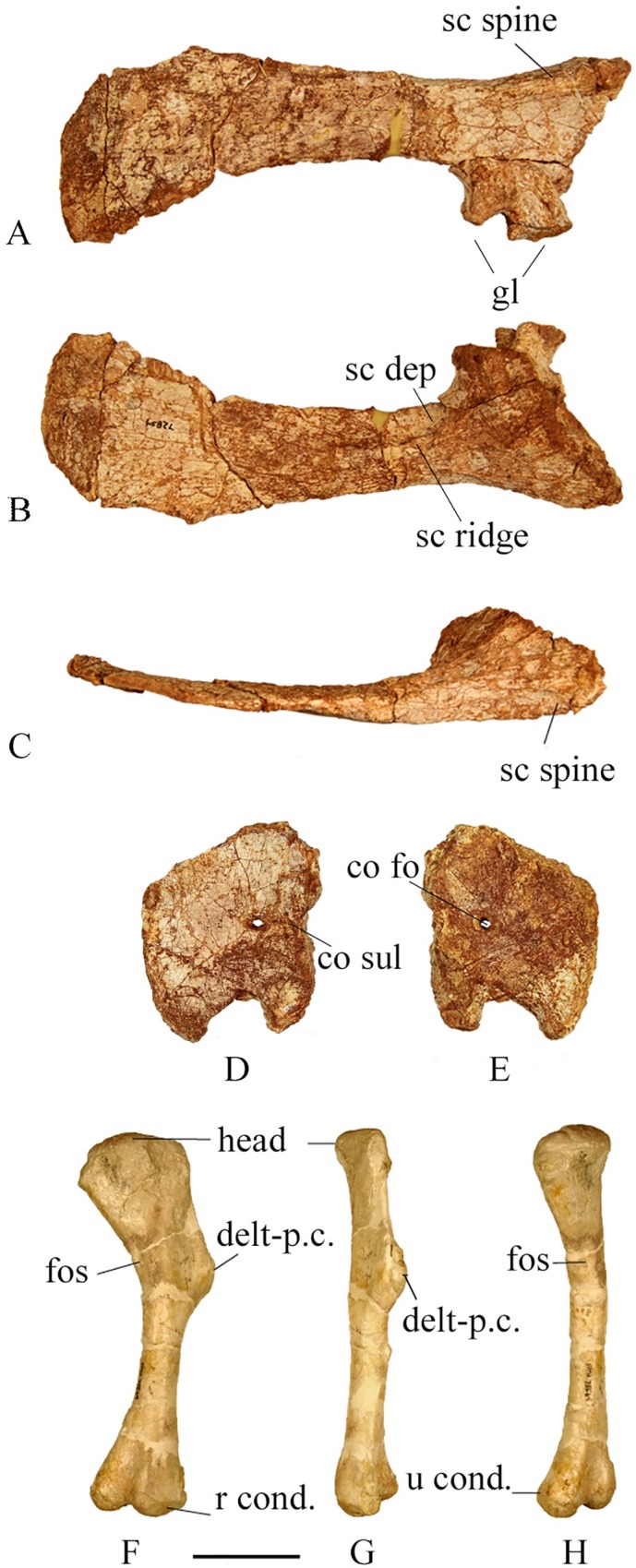
SMU 72834 scapula and coracoid. **SMU 75564 humerus.** (A) SMU 72834, right scapula in lateral view. (B) SMU 72834, right scapula in medial view. (C) SMU 72834, right scapula in dorsal view. (D) SMU 72834, right coracoid in medial view. (E) SMU 72834, right coracoid in lateral view. (F) SMU 75564, right humerus in posterior view. (G) SMU 75564, right humerus in lateral view. (H) SMU 75564, right humerus in medial view. Abbreviations: co fo-coracoid foramen, co sul- coracoid sulcus, gl-glenoid, sc dep- scapular depression, sc ridge- scapular ridge, sc spine-scapular spine, delt-p.c.-delta pectoral crest, fos-fossa, r. cond.-radius condyle, u cond.-ulna condyle. Scale bar equals 5 cm.

The medial surface of the scapular blade of the type specimen (SMU 72834, Fig 18) and a smaller specimen (SMU 71854) contains a shallow depression near the glenoid region that extends distally approximately halfway along the scapula. Its edge forms a prominent ridge along the anteromedial surface of the scapula. The dorsal and ventral edges of the scapula are round at the anterior end but become progressively thinner and sharper towards the posterior. The scapular blade becomes progressively thinner distally. The distal end of the scapular blade is expanded posteriorly forming a smooth and thin flaring crescent shaped end.

#### Coracoid

The coracoid is thicker at the scapular articular surface than more distally (Fig 18). The articulation surface with the scapula comprises the entire length of the proximal coracoid. The coracoid glenoid is slightly damaged in the type specimen, but smaller specimens preserve a smooth, broad coracoid glenoid that equally contributes to the glenoid cavity with the scapula glenoid. The circular coracoid foramen on the lateral surface is located close to the articular margin. On the medial surface the coracoid foramen is shifted closer to the articular margin and is oval shaped with a well-marked groove extending to the articular surface. This character has been noted in *Hypsilophodon foxii* [18], but is uncommon in basal ornithischians. In lateral view the coracoid is taller than wide in all specimens. The medial surface is concave; the lateral surface is slightly convex.

#### Humerus

The humerus is similar to other basal ornithischians. The best preserved humerus (SMU 75564) is from a larger sized individual, measuring 175 mm (Fig 18). Other preserved specimens range from 67–221 mm in length. The humerus is widest at the proximal end with the head being centered and bulbous. The distal shaft of the humerus is straight and circular in cross section. In lateral view the dorsal half of the humerus curves posteriorly beginning at the deltopectoral crest. The deltopectoral crest has a prominent groove running dorsoventrally along the anterior surface. The coronoid fossa is wider and more pronounced than the olecranon fossa. The medial condyle is round in ventral view and the lateral condyle is slightly larger with a tapered extension on the anterolateral corner.

Twelve complete or nearly complete humeri of range from 67 mm to 221 mm in length and presumably represent different ontogenetic ages allowing for a close inspection of ontogenetic change. The head of the humerus is more bulbous in larger individuals. The distal shaft of the humerus of smaller specimens is more oval in cross section becoming circular in larger individuals. The shafts are more twisted in larger individuals. A fossa is present on the medial surface of SMU 75564 opposite the deltapectoral crest. A shallow fossa is present on the larger type specimen (SMU 72834) as well, but is not as clearly defined.

#### Ulna

**Fig 19:**
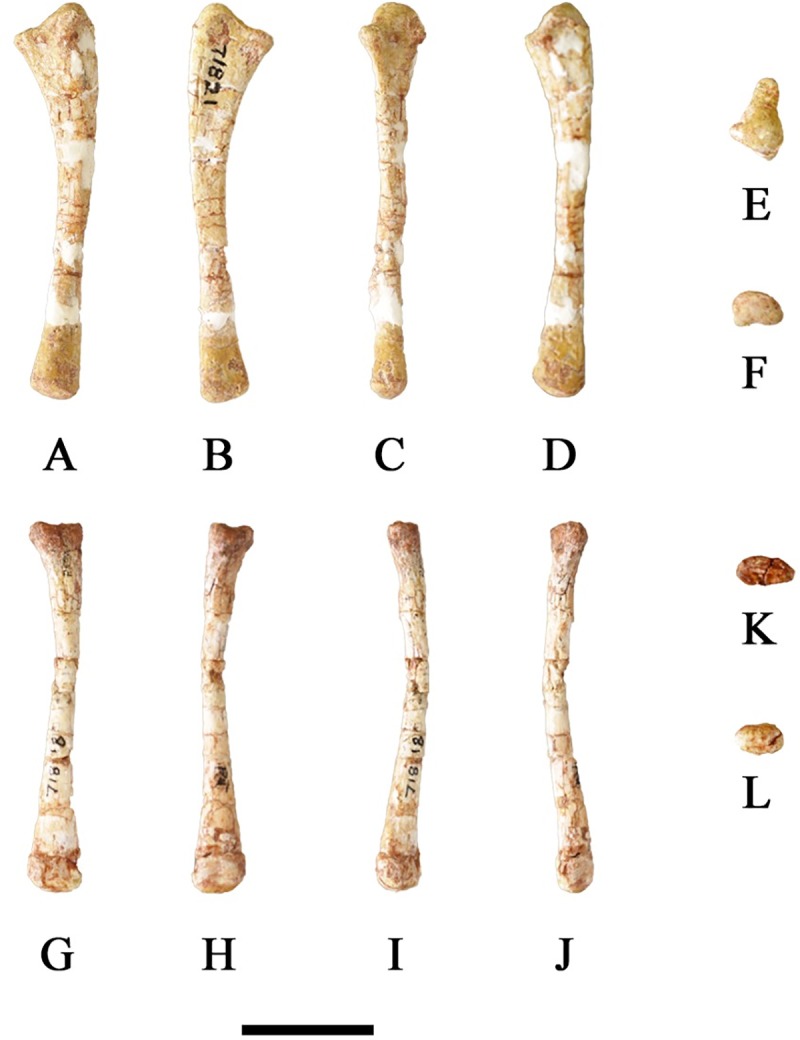
SMU 71821 ulna and SMU 71818 radius. (A) SMU 71821, left ulna in lateral view. (B) SMU 71821, left ulna in medial view. (C) SMU 71821, left ulna in posterior view. (D) SMU 71821, left ulna in anterior view. (E) SMU 71821, left ulna in proximal view. (F) SMU 71821, left ulna in distal view. (G) SMU 71818, left radius in lateral view. (H) SMU 71818, left radius in medial view. (I) SMU 71818, left radius in posterior view. (J) SMU 71818, left radius in anterior view. (K) SMU 71818, left radius in proximal view. (L) SMU 71818, left radius in distal view. Scale bar equals 2 cm.

The ulna is longer than the radius and the shaft is slightly bowed and medially concave (Fig 19). The cross-sectional shape of the shaft is oval. The lateral surface is round, but the medial surface is significantly flatter, especially the proximal and distal ends. The olecranon process is moderately developed with a concave radial articular surface on its proximolateral surface. The proximal end is triangular with the lateral half being larger than the medial half due to the presence of the olecranon process. The distal end of the ulna is slightly expanded and crescentic shaped.

#### Radius

The radius is approximately 90% the length of the ulna (Table 1). The proximal end is oval with a slightly concave articular surface for the humerus (Fig 19). The distal end is rounded with a smaller maximum diameter than the proximal end. Distinct ridges along the distal shaft impart a rounded “D” shape to the distal condyle. This differs from the flattened distal radius observed in *Tenontosaurus* [13].

#### Carpals

**Fig 20:**
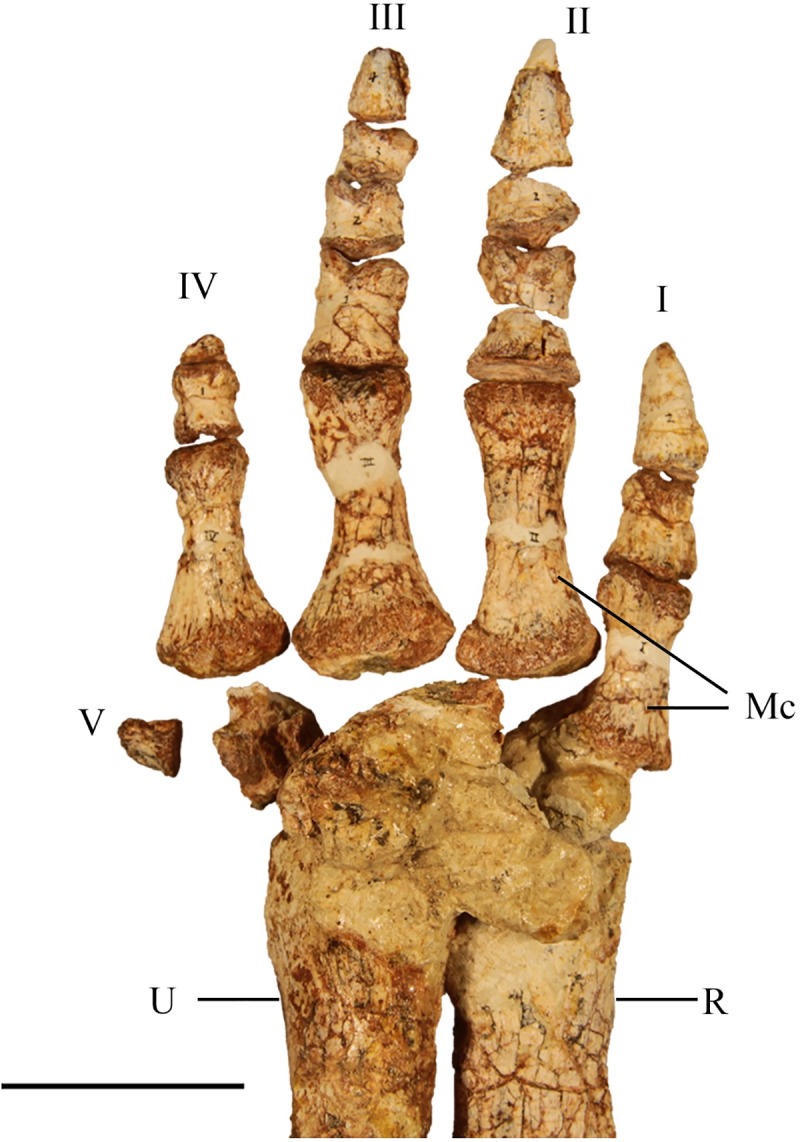
SMU 70456 left manus. Left manus (SMU 70456) in dorsal view. Abbreviations: I, II, III, IV, V- digits one through five, Mc-metacarpals, R-radius, U-ulna. Scale bar equals 5 cm.

SMU 70456 contains a complete left manus articulated to the radius and ulna; however, the carpals are damaged weathering limiting their description (Fig 20). A small round carpal is present distal to the ulnare. The ulnare is larger than the radiale and intermedium; however, descriptions on the exact shape cannot be given based on the available material.

#### Metacarpals

The articulated left manus of SMU 70456 contains five metacarpals with metacarpal III being the longest (Fig 20). The shape of metacarpals I, II, III, and IV is similar as each have expanded proximal and distal ends with the proximal end being larger. The shape and relative length of metacarpal V is unknown as only the distal end is preserved on SMU 70456. A shallow intercondylar groove is present on the ventral surface of the metacarpals.

#### Phalanges

The articulated left manus of SMU 70456 preserves a phalangeal formula of 2-3-4-2-1 (Fig 20). The first phalanges of each digit are the longest with each proceeding phalanx becoming progressively smaller until the ungual, forming ginglymoid articulations with one another. The unguals of digits I-III are claw like with pointed tips. The ungual of digit IV is significantly reduced. SMU 72054 contains a partial articulated left manus which shows digit III with only 3 phalanges, instead of four. Primitive taxa including *Hypsilophodon foxii* [18] and *Orodromeus makelai* [16] contain four phalanges on digit III whereas more derived taxa including *Tenontosaurus tilleti* [26] have three or fewer phalanges on digit III. The correct phalangeal formula for *Convolosaurus marri*, with noted polymorphism for digit III, is 2-3-(3,4)-2-1.

#### Ilium

**Fig 21:**
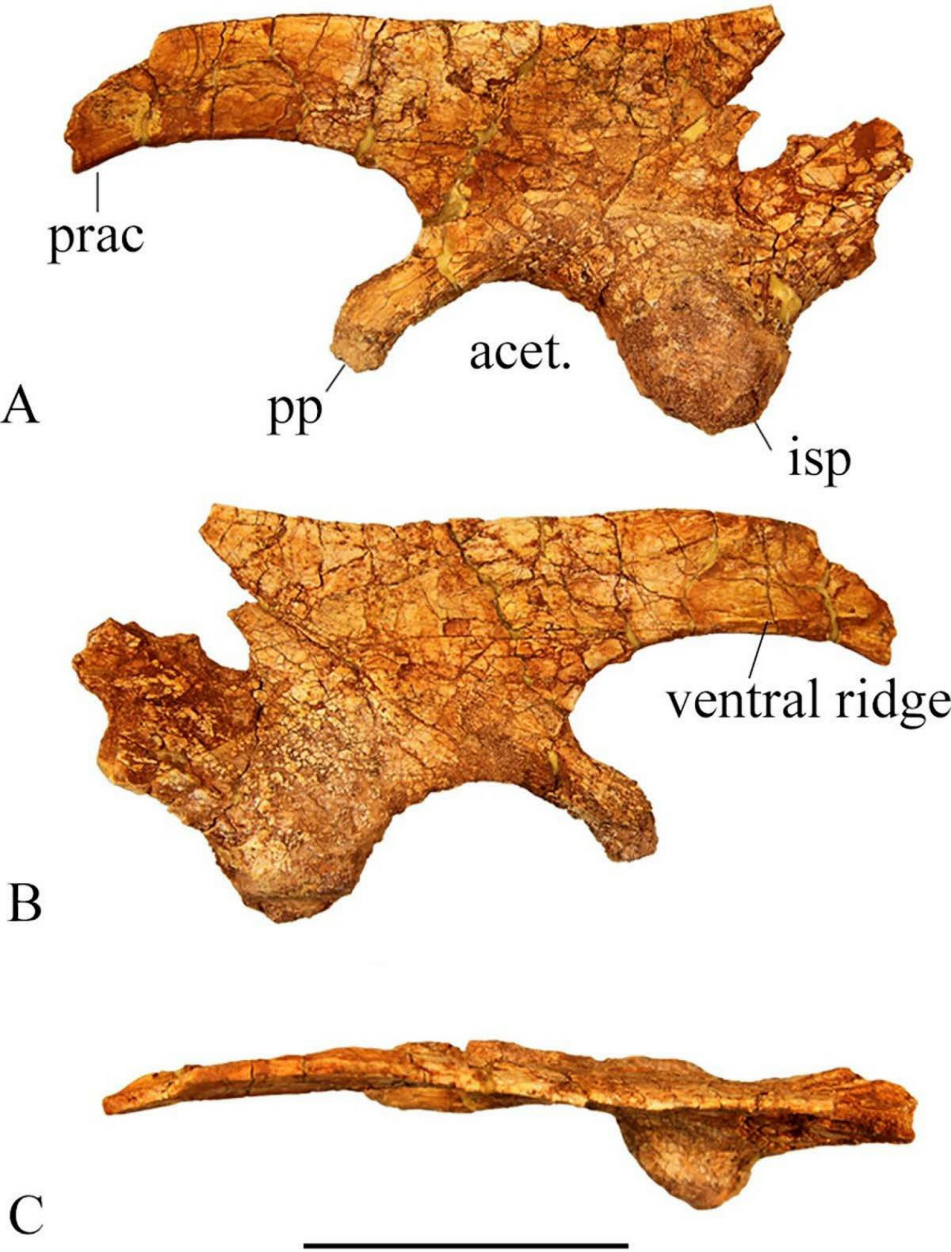
SMU 77617 left ilium. (A) Left ilium (SMU 77617) in lateral view. (B) Left ilium in medial view. (C) Left ilium in dorsal view with anterior end to the left. Abbreviations: acet.-acetabulum, isp- ischial peduncle, pp- pubic peduncle, prac- preacetabular process. Scale bar equals 5 cm.

The dorsal edge of the ilium is a uniformly thin blade anteriorly, but it thickens posteriorly. The sharp dorsal margin of SMU 70456 is straight, however a smaller partial ilium SMU 77617 (Fig 21) shows a sinuous dorsal margin. The pubic peduncle is transversely compressed and is shorter in length than the ischial peduncle. The pubic peduncle curves anteroventrally and tapers distally. The pubic peduncle is triangular in cross sections with a flat posterior surface for the acetabulum. The medial surface of the pubic peduncle contains a concave articular surface where the first sacral rib articulated to the ilium.

The lateral surface of the ilium is slightly concave. The ischial peduncle is robust and rounded forming the posterodorsal margin of the acetabulum. The round lateral boss of the ilium forms part of the synovial contact for the head of the femur. The ilium tapers posterior to the ischial peduncle as the ventral margin migrates dorsally ending with a squared posterior margin. A modest brevis shelf on the ilium extends medially posterior to the ischial peduncle. The preacetabular process tapers to a thin blade and deflects ventrolaterally. The medial margin is slightly concave with a weak ventral ridge.

#### Ischium

**Fig 22:**
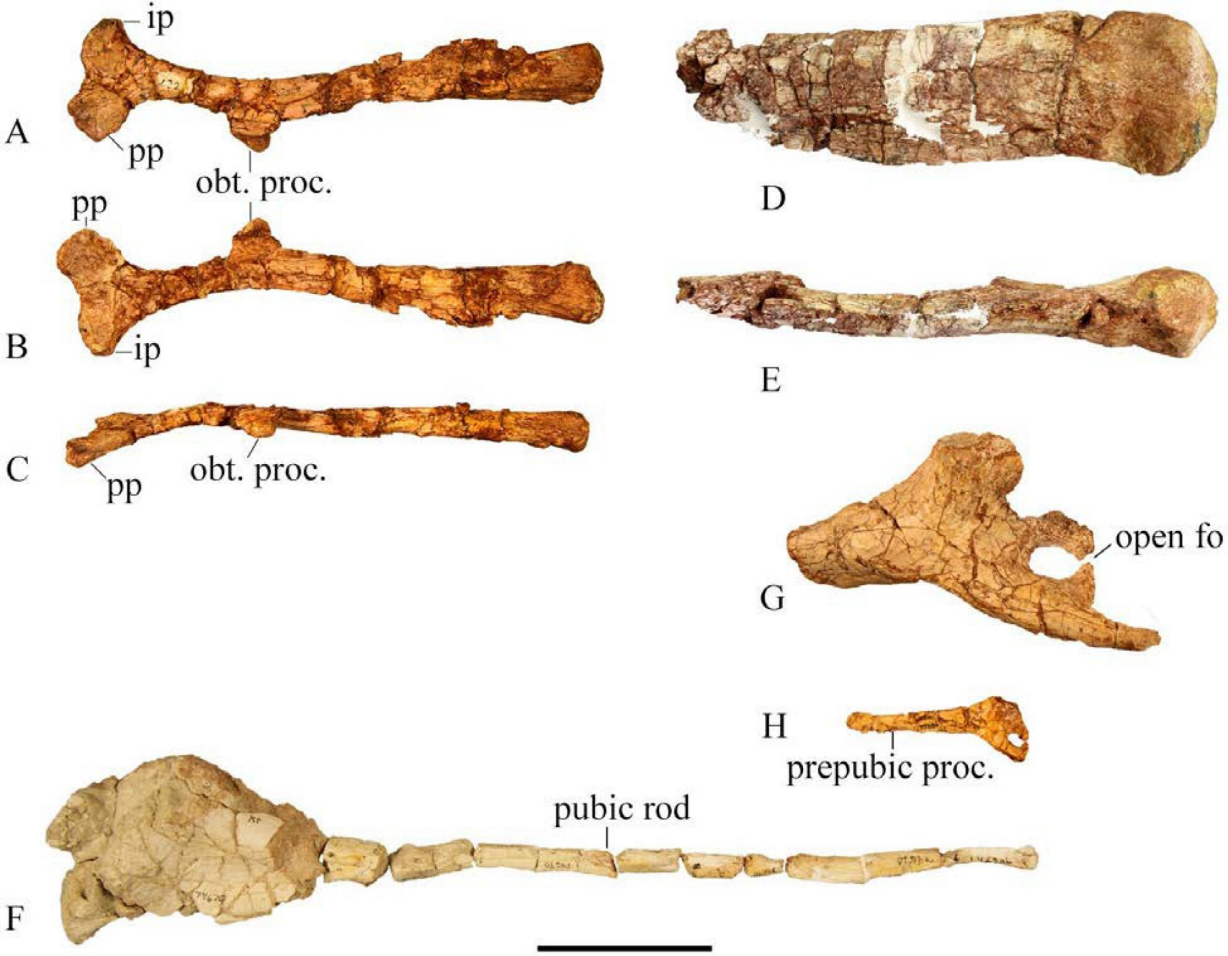
Ischium and pubis. (A) SMU 74119, right ischium in lateral view. (B) SMU 74119, right ischium in medial view. (C). SMU 74119, right ischium in ventral view. (D) SMU 77638, distal left ischium lateral view. (E) SMU 77638, distal left ischium dorsal view. (F) SMU 74679, left partial pubis in lateral view. (G) SMU 72316, left partial pubis. (H) SMU 75636, left partial pubis. Abbreviations: ip- iliac peduncle, obt. proc.- obturator process, open fo-open foramen, pp-pubic peduncle, prepubic proc.-prepubic process. Scale bar 5 cm.

A complete ischium preserved in SMU 74119 shows a flat elongated blade with a proximal region separated from the articular region by a constricted proximal shaft (Fig 22). The iliac peduncle is slightly thicker than the pubic peduncle and curves dorsomedially. The pubic peduncle is compressed mediolaterally and is larger than the iliac peduncle expanding anteroventrally with a slightly concave lateral surface. The obturator process falls in the proximal 1/3 of the shaft and curves ventrolaterally forming a concave articular surface for the rod-like pubis. In dorsal view the proximal half of the ischium is medially concave and the distal half is straight. The dorsal margin is rounded proximally, but progressively sharpens posteriorly. The ventral margin is sharp to the expanded flat distal surface. The lateral surface of the distal half of the ischium is slightly concave and expands medially. The distal end of the ischium is laterally expanded to form an ischial “foot” with a rounded and rugose posterior end (Fig 22D and 22E), but not flared as *Tenontosaurus* [13].

#### Pubis

No complete pubis has been recovered; however, SMU 74679, SMU 74104, SMU 72316, and SMU 75636 contain partial pubes (Fig 22). The prepubic process is straight in the smaller individual (SMU 75636), but curves dorsally in the larger specimen SMU 72316 similar to *Tenontosaurus* [13]. The prepubic process is laterally compressed anteriorly with a blunt, rounded distal tip that extends beyond the distal end of the preacetabular process of the ilium, distinguishing it from *Hypsilophodon foxii* [18]. The obturator region of the larger specimens SMU 72834 and SMU 72316 and the smaller SMU 75636 has an open foramen. However, the obturator region in SMU 74104 has a closed foramen. Individual variation in the obturator foramen has been noted in *Hypsilophodon foxii* [18] and *Orodromeus makelai* [16]. The postpubic process is straight and rod-shaped progressively tapering and becoming laterally compressed distally. The dorsal portion of the main body of the pubis is robust and rounded. The articular surface for the ilium is slightly concave.

#### Femur

**Fig 23:**
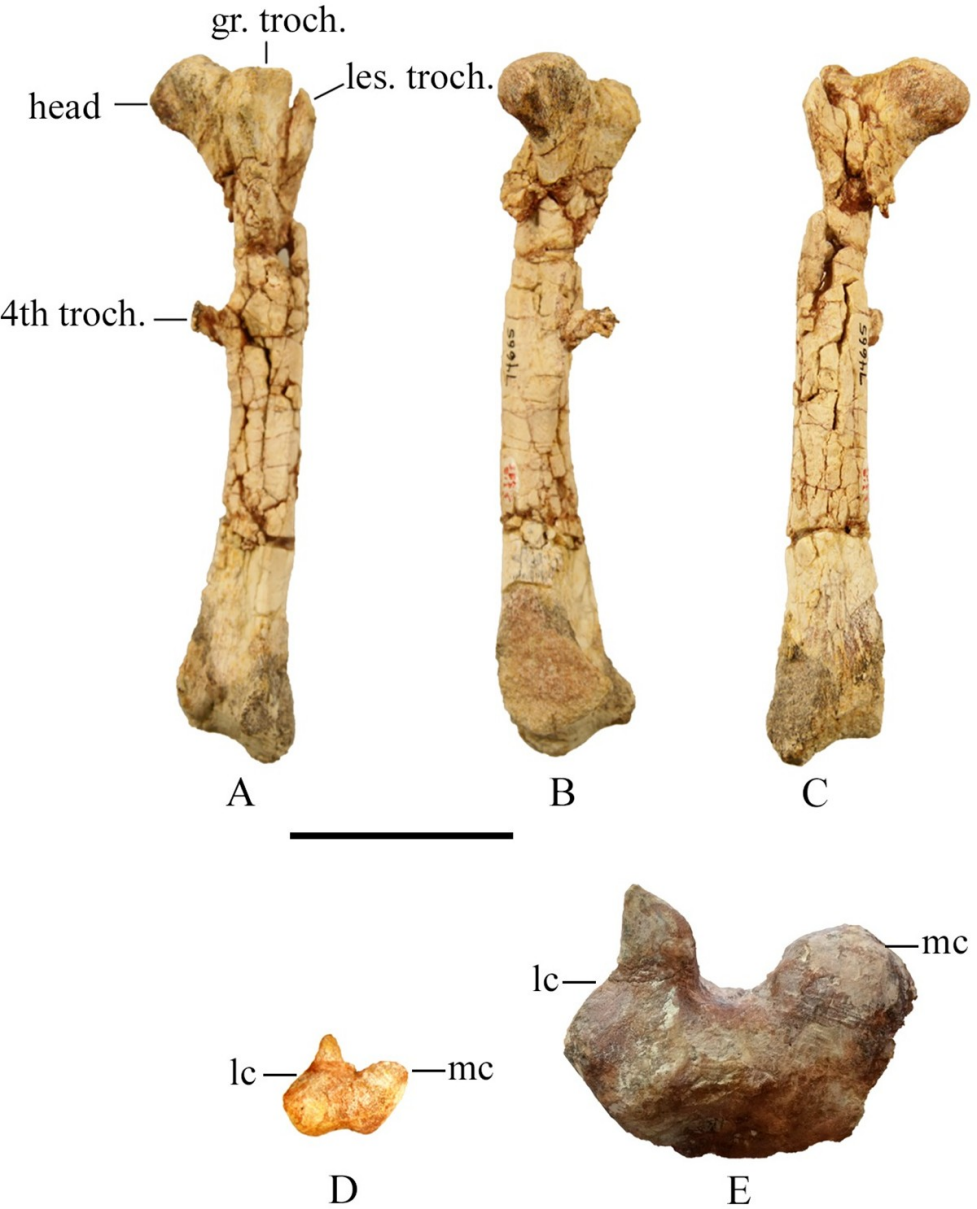
Femora. (A) SMU 74665, right femur in posterior view. (B) SMU 74665, right femur in medial view. (C). SMU 74665, right femur in anterior view. (D) SMU 72451, left femur ventral view. (E) SMU 70456, left femur ventral view. Abbreviations: gr. troch.- greater trochanter, lc-lateral condyle, les. troch.- lesser trochanter, mc-medial condyle, 4th troch.- fourth trochanter. Scale bar 5 cm.

Femur length ranges from 51 mm to 315 mm, capturing a range of ontogenetic size classes. The shaft of the femur is straight and twists slightly in the posterior half such that the lateral surface is more anteriorly facing (Fig 23). In lateral view the femur is bowed anteriorly along its length. The proximal end of the femur contains the lesser trochanter on the anterolateral surface which is subtriangular at its dorsal margin. The greater trochanter is separated from the lesser trochanter by a narrow v-shaped groove. The lateral surface of the greater trochanter is fairly flat, but its dorsal margin is a rounded crest which extends further than the lesser trochanter. The head is angled approximately 20 degrees from the transverse plane extending the dorsal margin of the femoral head above the level of the greater trochanter. The posterior face of the femoral head is concave with a strong depression running diagonally across its surface to the neck of the femur.

**Fig 24:**
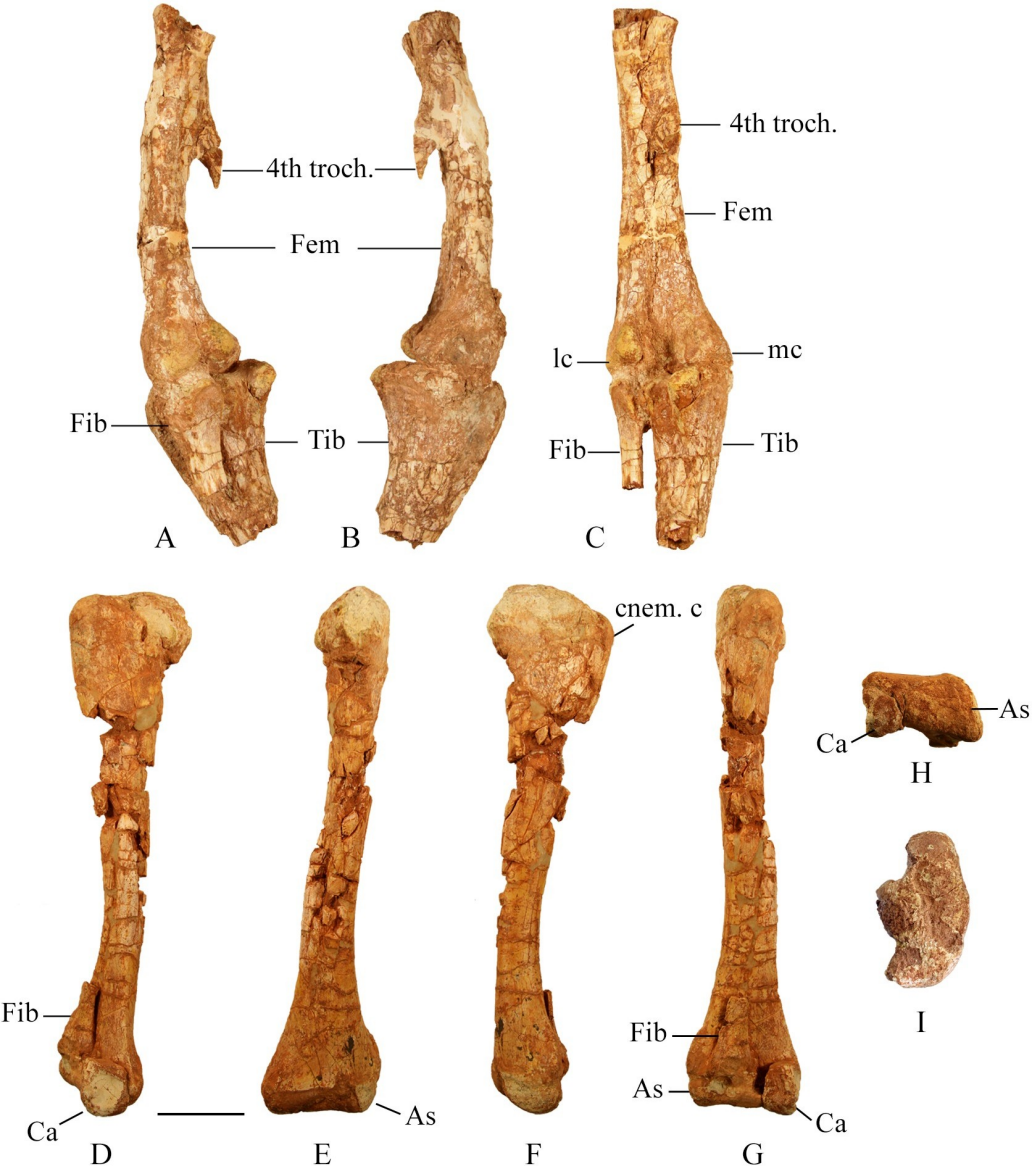
Hind limb. (A) SMU 72316, left hind limb in lateral view. (B) SMU 72316, left hind limb in medial view. (C). SMU 72316, left hind limb in posterior view. (D) SMU 77617, right tibia, fibula, astragalus, calcaneum in lateral view. (E) SMU 77617, right tibia, fibula, astragalus, calcaneum in posterior view. (F) SMU 77617, right tibia, fibula, astragalus, calcaneum in medial view. (G) SMU 77617, right tibia in anterior view. (H) SMU 77617, right tibia in ventral view. (I) SMU 72834, proximal left tibia in dorsal view. Abbreviations: As- astragalus, Ca- calcaneum, cnem. c- cnemial crest, Fem-femur, Fib- fibula, lc-lateral condyle, mc-medial condyle, Tib-tibia, 4th troch.- fourth trochanter. Scale bar 5 cm.

The fourth trochanter is located on the medial margin of the proximal half of the femur. It consists of a pendant shaped blade that extends posteroventrally and has a thick ventral edge that thins posteriorly (Fig 24). The fourth trochanter is larger more prominent in larger specimens. A shallow depression occurs on the medial surface of the femoral shaft posterior to the femoral neck and extending to the base of the fourth trochanter. Galton (1974) identified this as the insertion for the *M*. *caudifemoralis longus*. The minimum circumference of the femoral shaft is just distal to the fourth trochanter. The cross section is circular in smaller individuals, but subtriangular in larger individuals. The distal portion of the shaft expands mediolaterally towards the condyles. The medial condyle is rounded and more robust than the lateral condyle. The lateral condyle tapers posterolaterally forming a ‘teardrop’ shape in ventral view that extends posteriorly further than the medial condyle. Posteriorly the condyles are separated by a deep intercondylar groove. Anteriorly a shallow distal extensor groove separates the condyles. The extensor groove is more pronounced in larger specimens.

#### Tibia

The tibia in SMU 71836 is approximately equal in length to the femur measuring 100 mm compared to the femur measuring 99 mm. In larger specimens including SMU 74093 and SMU 74670 the tibia is approximately 15–20% longer than the femur. In all specimens the tibia has a narrower shaft than the femur. In anterior view the shaft of the tibia is sigmoidal as the lateral edge is slightly concave proximally and slightly convex distally. The medial edge is slightly convex proximally and concave distally (Fig 24). Proximally the tibia has a dual-lobed lateral condyle and a smaller medial condyle. The anterolateral surface of the lateral malleolus forms the articulation surface for the fibula, and the posterior condyle forms a slight ridge extending a short distance along the shaft. The small cnemial crest is rounded and extends anterolaterally along the shaft for a short distance.

In cross section the tibia is roughly triangular in shape with rounded corners in the proximal half. The distal half contains a pointed corner created by a sharp lateral edge which begins at the lateral malleolus. The posterior surface of the distal end contains attachment and muscle scars as seen in *Hypsilophodon foxii* [18], *Zephyrosaurus schaffi* [17], and *Orodromeus makelai* [16]. Attachment scars found on the anterior surface of the lateral edge represent the distal articulation surface for the fibula. There is a very shallow ligament groove on the anterior surface of the distal end which forms the articular surface of the ascending process of the astragalus and separates the malleoli. In ventral view the medial malleolus is slightly anteroposteriorly expanded and the lateral malleolus is compressed anteroposteriorly and contains a sharp lateral edge.

#### Fibula

The medial surface of proximal head of the fibula is concave as it articulates with the tibia, but gradually flattens to the neck. A thick neck supports the proximal end of the fibula which then tapers distally to a thin fibular shaft. The diameter of the shaft decreases distally which is also marked by a change in cross sectional shape. The shaft of the proximal third of the fibula is D-shaped in cross section with a flat medial surface. This transitions into a circular rod-shaped shaft in the middle third of the fibula which transition again into a D-shape in the distal third as the posterior surface becomes flat creating an articulation surface with the lateral malleolus of the tibia. The distal end of the fibula has as an anterior expansion with a flat ventral surface that articulates with the calcaneum.

#### Astragalus

**Fig 25:**
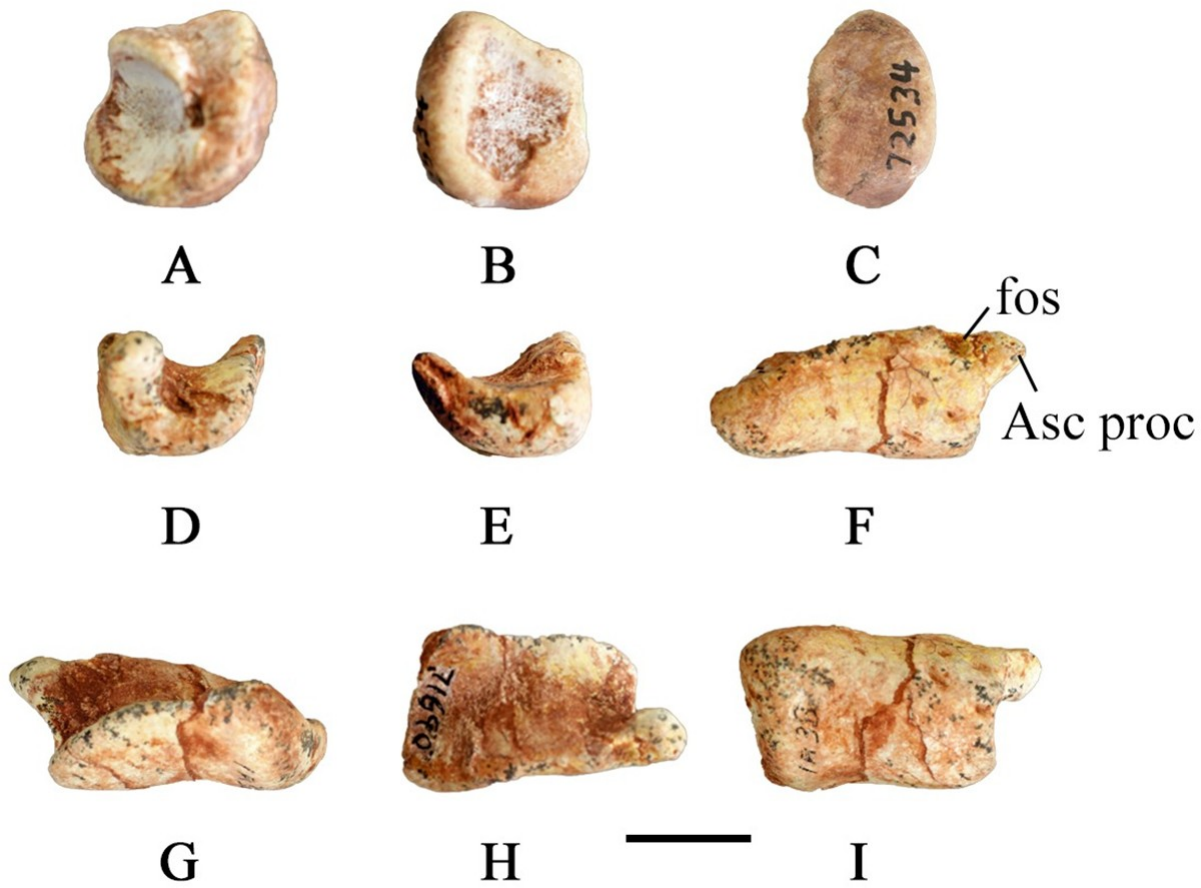
Astragalus and calcaneum. (A) SMU 72534, left calcaneum in medial view. (B) SMU 72534, left calcaneum in lateral view. (C). SMU 72534, left calcaneum in anterior view. (D) SMU 71690, left astragalus in medial view. (E) SMU 71690, left astragalus in lateral view. (F) SMU 71690, left astragalus in anterior view. (G) SMU 71690, left astragalus in posterior view. (H) SMU 71690, left astragalus in dorsal view. (I) SMU 71690, left astragalus in ventral view. Abbreviations: Asc proc- astragalus ascending process, fos-fossa. Scale bar 1 cm.

Several astragali are preserved; however, the ascending process on the larger specimens is missing, thus smaller specimens are used in the description. SMU 71690 represents a complete left astragalus (Fig 25). In ventral view the astragalus caps the ventral surface of the medial malleolus of the tibia and small portions of the lateral malleolus. The astragalus then curves and extends dorsally over the anterior surface of the medial malleolus of the tibia. This ascending process of the astragalus tapers dorsolaterally similar to *Hypsilophodon foxii* [18]. On the anterodorsal surface just below the ascending process is a fossa which is not present in *Hypsilophodon foxii* [18] (Fig 25). The medial surface of the astragalus is concave forming the articulation surface with the calcaneum.

#### Calcaneum

SMU 72534 represents a complete calcaneum for this taxon (Fig 25). The posteromedial surface of the calcaneum articulates to the anteromedial surface of the lateral malleolus of the tibia. The dorsal surface is concave creating a cup which articulates with the distal end of the fibula. A slight notch is located on the medial surface which articulates with the astragalus. The lateral surface of the calcaneum is also strongly concave. The anterior surface is smooth and convex. The main articulation surface for the astragalus is fairly flat.

#### Medial distal tarsal

SMU 70534 contains an articulated left hind limb including articulated medial and lateral distal tarsals. The medial distal tarsal is a square blocky bone with rounded corners and edges except for a sharp medial edge. The dorsal surface is irregularly concave in the center, but convex dorsomedially. Dorsolaterally it forms an articulation surface with the astragalus. The posteroventral surface articulates with the proximal end of metatarsals II and III. The depression formed by a beveled ventromedial corner observed in *Hypsilophodon foxii* [18] is not observed in *Convolosaurus marri*. Its lateral edge is slightly concave forming an articular surface with the lateral distal tarsal.

#### Lateral distal tarsal

The lateral distal tarsal is wedge-shaped bone as its anterior end is relatively thin and the proximal ends are expanded dorsoventrally. Its dorsal surface is concave forming an articular surface with the calcaneum. The medial surface is slightly convex articulating with the astragalus. The ventral surface is also slightly convex articulating with the concave dorsal surface of metatarsal IV. Metatarsal V likely articulated with the ventral surface of the lateral distal tarsal; however, it is disarticulated in SMU 70534.

#### Metatarsals

**Fig 26:**
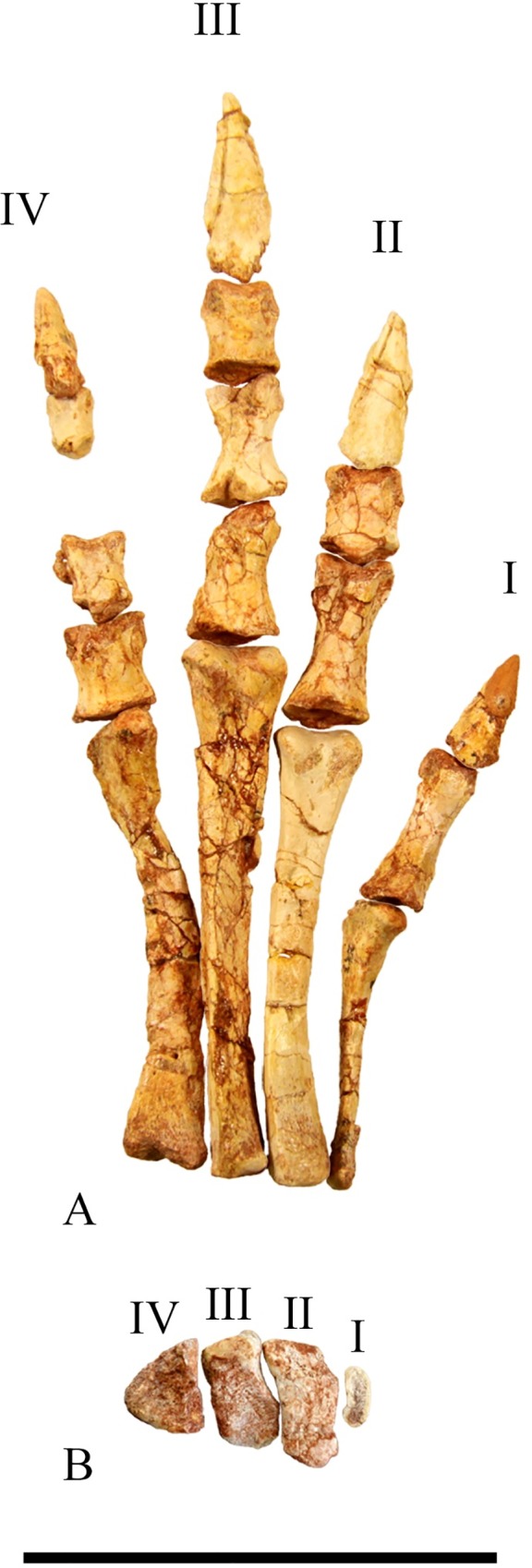
Pes. (A) SMU 77636, left articulated pes in dorsal view. (B) SMU 77636, left articulated metatarsals in proximal view. Abbreviations: I, II, III, IV- digits one through four. Scale bar equals 3 cm.

SMU 70534 and SMU 77636 (Fig 26) are semi-articulated left feet and SMU 73170 is a smaller articulated left foot. The proximal ends of metatarsals I and II are expanded anteroposteriorly, whereas the distal ends are expanded mediolaterally giving them a slightly twisted appearance. Metatarsal I is approximately half the length of metatarsal III with a concave medial surface (Table 1). The shaft of metatarsal I is laterally compressed, but distally expands to form a robust distal condyle. At midshaft it is elliptical in cross section and distal portions of the shaft are triangular in cross section. The dorsal surface of metatarsal I is concave with a sharp edge in the proximal half that extends and widens in the distal half. The ventral surface also has a sharp edge in the proximal half that flattens becoming slightly concave in the distal half of the metatarsal. The flattened lateral surface of metatarsal I articulates to the posteromedial surface of metatarsal II. The distal end of metatarsal I is comprised of a robust condyle with no trochlea.

Metatarsal II is slightly shorter than metatarsal III and equal in length to metatarsal IV. The proximal half is laterally compressed with a triangular cross section, whereas the distal half is strongly compressed anteroposteriorly creating an elliptical cross-sectional shape. In dorsal view the medial side is slightly concave and the lateral side is straight. The medial surface of metatarsal II is flat to slightly convex. The dorsal surface is fairly flat with a slight depression in the distal portions which extends into the intercondylar groove. The lateral surface is flat and articulates to the medial surface of metatarsal III. The distal end of metatarsal II is comprised of two condyles, the medial condyle being slightly larger than the lateral. The outer surface of each condyle is marked by ligament pits, but the medial pit is more prominent.

Metatarsal III is the longest metatarsal and is similar in morphology to metatarsal II, but its proximal half is less laterally compressed and the distal portion is dorsoventrally compressed. Its medial surface is broad and flat on the proximal end, but it then becomes rounded and thinner distally. The proximal surface is slightly sigmoidal. The dorsal surface is fairly flat with a shallow extensor groove in the distal portion that forms an intercondylar groove on the distal end. The ventral surface is slightly convex in the proximal half and distally forms a flat surface that also contains a shallow extensor groove. The lateral surface is flat and broad in the proximal half, but this surface becomes rounded in the distal half. The distal end consists of two condyles the medial being more robust but the lateral extends slightly farther. The outer surface of each condyle is marked by ligament pits, but the medial pit is more prominent.

Metatarsal IV is equal in length to metatarsal II and has the widest proximal end of any metatarsal which is triangular in dorsal view. In dorsal view the medial edge is slightly concave and the lateral edge is flat. The lateral side has a prominent rounded ridge that diminishes distally forming a flat surface. The dorsal surface is flat to slightly convex. The distal end consists of a single condyle that contains a shallow extensor groove on the ventral surface and a ligament pit on its lateral surface. The distal surface of metatarsal IV is slightly rounded.

Metatarsal V is a reduced splint found articulated to the posterolateral side of metatarsal IV. SMU 70534 contains metatarsal V; however, it is not in its original articulation. Based on articulation in other basal ornithischians it likely articulated to the ventral surface of the lateral distal tarsal. Metatarsal V is approximately 34% the length of metatarsal III in this specimen. No phalanges were found associated with metatarsal V.

#### Phalanges

The phalangeal formula for the pes of this taxon is 2-3-4-5-0 (Fig 26). The first phalanges of each digit are the longest with each proceeding phalanx becoming progressively smaller until the ungual. The proximal end of each phalanx is concave and the distal end contains two condyles with prominent extensor grooves on the dorsal and ventral surface as well as defined collateral ligament pits. Deep grooves mark both the anteromedial and anterolateral surface of the unguals. Claw like unguals are present in digits I-IV and are longer than they are wide with flattened but proximodistally curved ventral surfaces. The unguals of the larger and presumably ontogenetically older SMU 70534 are proportionally wider than the unguals of the smaller and presumably younger individual SMU 73170 and SMU 73171.

## Ontogeny and behavior

**Fig 27:**
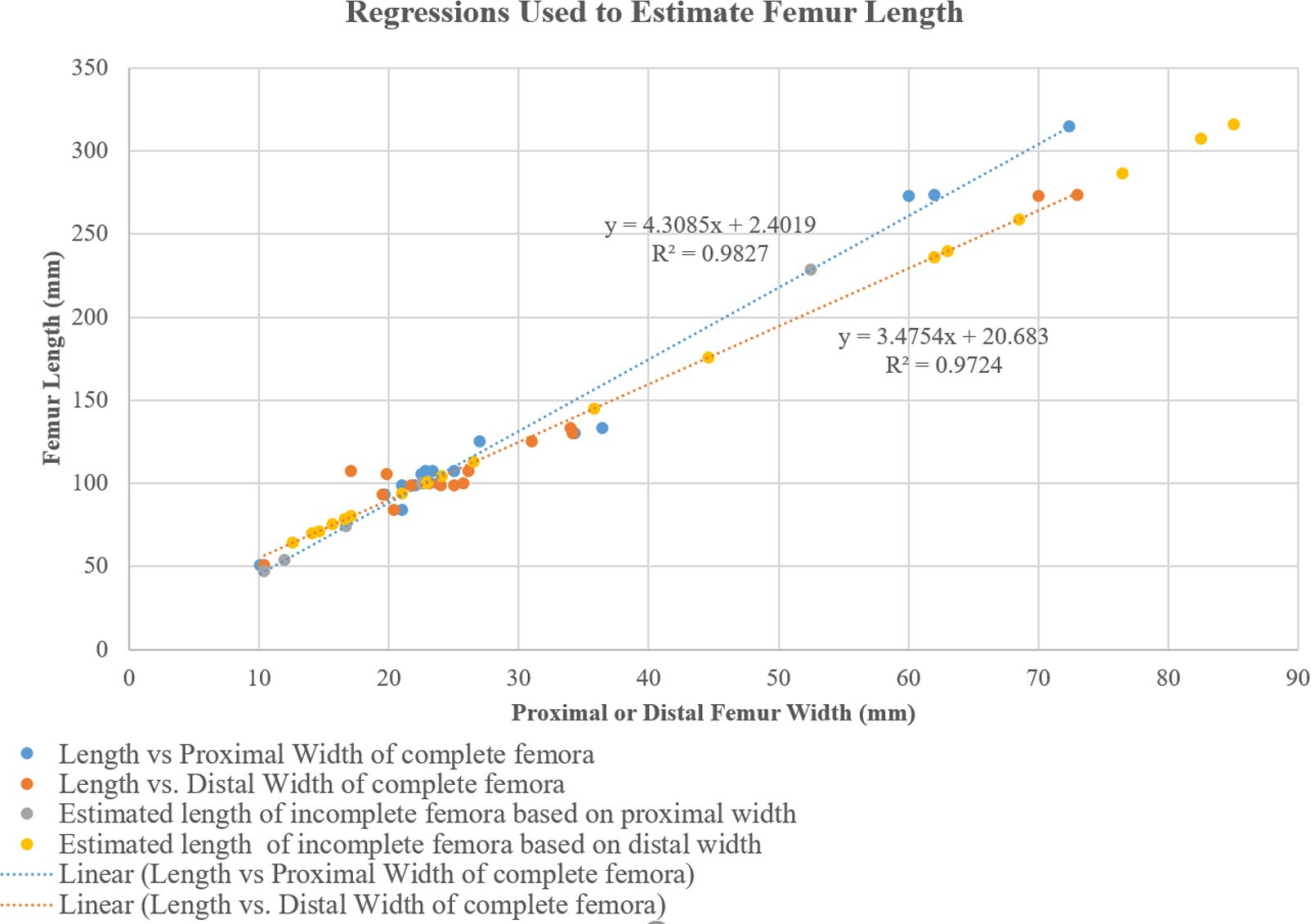
Femur size distribution from Proctor Lake. Scatter plot of femur length versus proximal and distal width from Proctor Lake. Nineteen complete femora were used to create two linear regression models which was then used to estimate the length of 26 additional partial femora based on proximal and distal width. Measurements listed in S1 Table.

**Fig 28:**
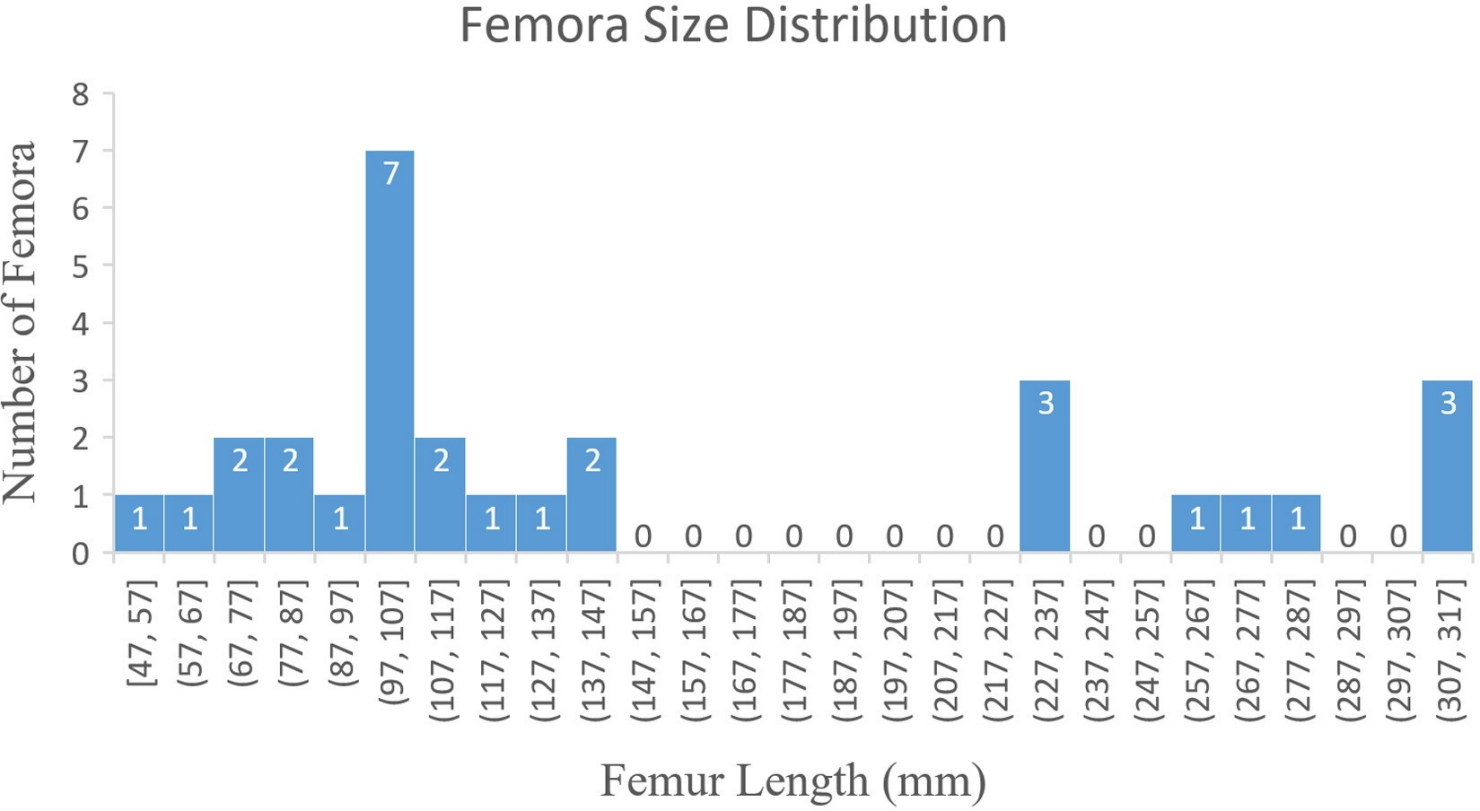
Histogram of femur length distribution from Proctor Lake fossil locality. Histogram of femur length distribution from Proctor Lake fossil locality. Graph displaying range of femoral length of 29 individuals recovered from the Proctor Lake locality.

The Proctor Lake fossil locality contains a minimum of 29 individuals ranging in size and ontogenetic stages. For example, femur length ranges from 51 mm to 315 mm and tibia length ranges from an estimated 64 mm to 376 mm. Nineteen complete femora were used to estimate femur length for an additional 26 incomplete femora based on linear regression models developed for proximal and distal width of the femora (Fig 27; S1 Table). The high R-squared values of this model support the interpretation that the individuals recovered from Proctor Lake locality represent a single species. The Proctor Lake locality is dominated by smaller and ontogenetically younger individuals with few larger individuals, suggesting that the Proctor Lake locality could possibly be a nesting site (Fig 28). The femur size distribution indicates a higher mortality rate in younger individuals corresponding to results from Woodward et al. (2015) analyzing population dynamics of the hadrosaurid dinosaur *Maiasaura peeblesorum*. This result seems plausible considering younger and weaker individuals may have been more susceptible during crisis scenarios such as drought. No evidence appears in the taphonomic or skeletal analyses to suggest these individuals died as a result of predation. Such evidence includes lack of tooth marks on any of the observed *C*. *marri* skeletal material and only one shed dromaeosaur tooth (SMU 70435) recovered among the hundreds of specimens of *C*. *marri*.

**Fig 29:**
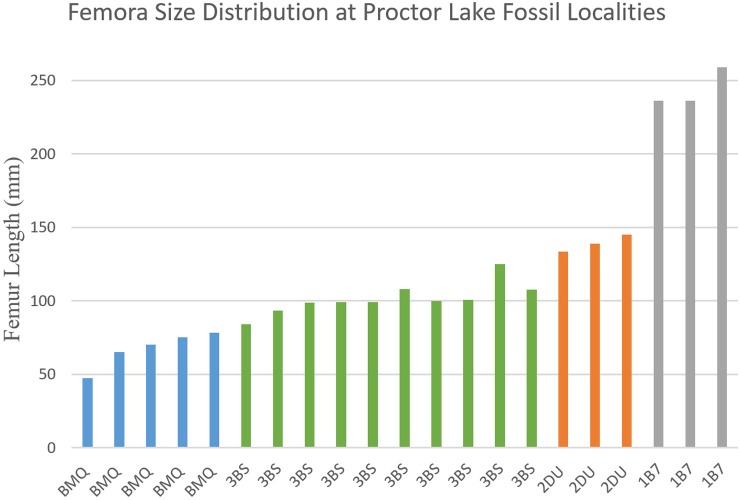
Histogram of femur length distribution from Proctor Lake locality fossil sites. Graph displaying range of femoral length of individuals found in clusters. Site localities are listed along the horizontal axis.

Four of the fossil localities at Proctor Lake contain clusters of individuals that are partially articulated; however, the average size of individuals in each cluster differs (Fig 29). Localities 3BS and 2DU are located in the North Quarry with locality 3BS located 14 meters west of locality 2DU. Localities BMQ and 1B7 are located in the Camp Quarry with locality BMQ located 44 meters northeast of 1B7. Detailed notes of these localities are stored with the fossils in the Shuler Museum of Paleontology at SMU. The clusters are semi-articulated individuals, many of which are superimposed [1] (S1 Fig). There is no preferred orientation of bones or separation by size or density which suggests these clusters indicate behavioral aspects of the individuals rather than post depositional sorting by fluvial events [1] (S1 Fig). This is further supported by the sedimentology at each locality which consists of well-developed vertic paleosols with no evidence of major fluvial influence. At site BMQ a minimum of five individuals are clustered with an average femora length of 67 mm. Femora from a cluster of 10 individuals recovered from 3BS average 102 mm in length. The femora of three individuals clustered at site 2DU average a length of 139 mm and femora from three individuals clustered at 1B7 average 244 mm. Individuals preserved at sites BMQ and 3BS preserve features of younger ontogenetic stages including approximately equal femur and tibia lengths and fewer dentary teeth (S2 Table). Individuals preserved at sites 2DU and 1B7 preserve more developed features including larger tibia relative to the femur, larger scapula relative to the humerus, and increased number of maxillary and dentary teeth (S2 Table). This indicates individuals grouped together after hatching and may have flocked together for protection from predators [1; 28].

Histological analysis of four femora recovered from the Proctor Lake locality was conducted by Winkler (1994) in an effort to determine the ontogenetic stage and growth rate of this taxon. Studies of bone histology has been employed to understand the growth of several ornithischian species including *Dryosaurus lettowvorbecki*, *Orodromeus makelai*, *Dryosaurus altus*, *Tenontosaurus tilletti*, and *Maiasaura peeblesorum* [30; 31; 32; 27]. In extant vertebrates LAGs (lines of arrested growth) represent temporary arrested growth and have been shown to have annual periodicity [33; 34; 35]. This concept has been applied extensively to the fossil record including the aforementioned ornithischian taxa to estimate ontogenetic age of individuals. Histological analysis conducted on a large femur recovered from Proctor Lake did not reveal any LAGs, although secondary osteons are present [29] (S2 Fig).

The absence of LAGs in one of the largest femora recovered from the Proctor Lake locality might suggest the individuals recovered are less than one year old or that growth was rapid and continuous in this dinosaur. However, given the size distribution of femora recovered and correlated with differences observed in limb proportions and tooth count, it seems unlikely that the individuals are all less than one year old. This is also supported by the tibia size distribution recovered by Woodward et al. (2015) for the hadrosaurid *Maiasaura*, which recovered a high concentration of juvenile individuals followed by low concentrations of subadult and adult individuals all of whom were at least two years old. If similar population dynamics are assumed for the Proctor Lake locality, the larger individuals recovered represent subadult or adult ontogenetic stages. The search for LAGs is hampered by the poor microstructure preservation, especially of the external fundamental system in many larger specimens from Proctor Lake. Similarly, histological analyses of *Dryosaurus lettowvorbecki*, *Dryosaurus altus*, and an Early Cretaceous unnamed hypsilophodontid recovered from Australia does not show LAGs in the largest sampled elements [30; 32; 36]. Chinsamy (1995) suggested the lack of LAGs in *Dryosaurus lettowvorbecki* reflects an indeterminate growth pattern, differing from many ornithischian taxa. Given the phylogenetic distribution of determinate growth patterns in closely related taxa, Horner et al. (2009) argued that the lack of LAGs in *Dryosaurus lettowvorbecki* and *Dryosaurus altus* reflected an absence of adult specimens, which could have implications for their positioning within ornithischian phylogeny. The pattern of growth in *C*. *marri* may be similar to *Tenontosaurus* in that growth was rapid to sub-adult size, followed by much slower growth rates [37].

## Phylogenetic analysis

Morphological characters of *C*. *marri*, were scored using a modified matrix from Baron et al. (2016) which expanded on datasets compiled by Butler et al. (2007), Butler (2010), Butler et al. (2011), Han et al. (2012), Ősi et al. (2012), and Barrett et al. (2014) [39; 40; 41; 42; 43; 44] (S1 Text and S3 Table). Modifications included adding five additional characters (#228, #229, #230, #231, #232 (Scheetz 1999), one new character (#233: presence of median sulcus on buccal surface of premaxillary teeth), rescoring 11 characters for *T*. *dossi* (S4 Table), and adding *C*. *marri*. As in previous analyses, five unstable ‘wild card’ taxa including *Yandusaurus*, *Anabisetia*, *Echinodon*, *Yueosaurus*, and *Koreanosaurus* were excluded from the final data set creating a matrix of 50 taxa and 233 characters. The matrix was compiled and edited using Mesquite v.2.74 [45]. The matrix was analyzed using TNT 1.5 [46] with all characters being treated as unordered except characters #112, #135, #137, #138, and #174 following previous studies [38; 41; 42; 43; 44]. The search was run using 1000 replications of Wagner trees (with random addition sequence) followed by a tree bisection reconnection (TBR) swapping algorithm (holding 10 trees per replicate). Zero-length branches were collapsed on all recovered most parsimonious trees. Bootstrap analysis was performed using TNT for 1000 replicates (each using a heuristic search of 100 replicates) Bremer support was also calculated using TNT.

## Results

**Fig 30:**
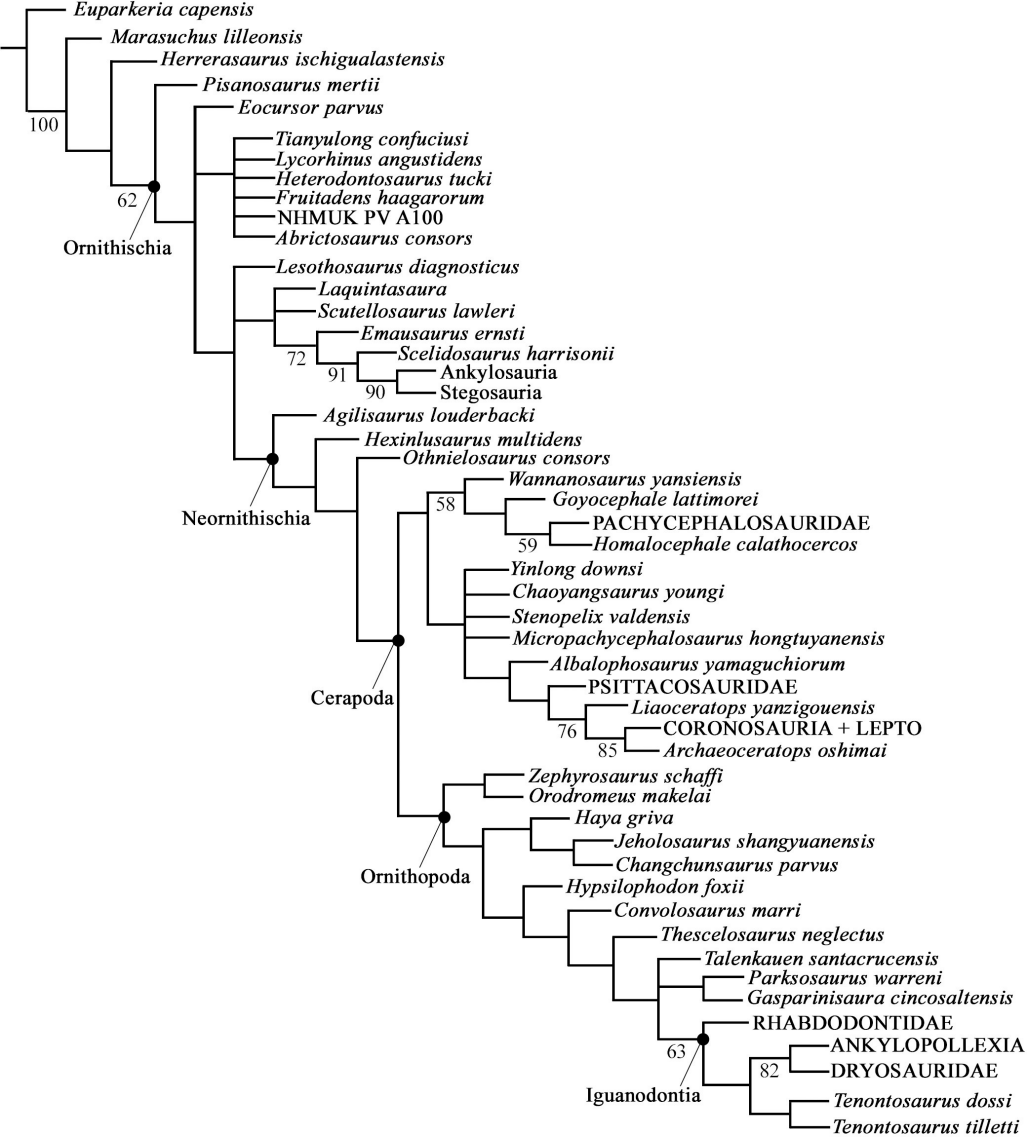
Strict consensus tree produced from phylogenetic analysis. Strict consensus tree of 96 most parsimonious trees recovered from phylogenetic analysis. Bootstrap support values >50% listed beneath nodes.

The analysis of the modified matrix retained 96 most parsimonious trees with a best score of 607. The strict consensus tree recovers *C*. *marri* as sister to a clade containing *Thescelosaurus neglectus* and Iguanodontia (Fig 30). *Hypsilophodon foxii* is recovered sister to a clade containing *C*. *marri*, *Thescelosaurus neglectus* and Iguanodontia (Fig 30). Autapomorphies recovered for *C*. *marri* include the presence of four premaxillary teeth (112:2), opisthocoelous cervical vertebrae (134: 1); convergently present in *Agilisaurus louderbacki* [23] and Iguanodontia, an expanded ischial ‘foot’ (182:1); convergently present in *Heterodontosaurus tucki* [47] and members of Iguanodontia, and the presence of a medial sulcus on the buccal surface of the premaxillary teeth (233:1). The monophyletic group sister to *Hypsilophodon foxii* [18] is unambiguously united in possessing curved maxillary tooth roots (230: 1), sacral neural spines at least twice the height of the sacral centra (144: 1 and 2), and proximal caudal spines at least 1.5 times the height of the caudal centra (142: 1; convergently present in Stegosauria). Iguanodontia is supported unambiguously by possessing diamond shaped maxillary and dentary crowns (115: 2), the absence of cingulum on the maxillary and dentary teeth (123:1); convergently present in *Heterodontosaurus tucki* [47], and a laterally inflated medial condyle which partial covers the opening of the flexor (intercondylar) groove of the femur (204: 1).

## Discussion

Ornithopods are a diverse group with numerous character reversals and parallelisms which makes the interpretation of their evolution difficult. The phylogeny of basal ornithischians in particular is poorly resolved and relationships are weakly supported as evidenced by recent studies [38; 48]. This can be attributed in part to the fragmentary nature of many of the taxa making the discovery of the nearly complete skeletons of *C*. *marri* crucial to aid in the understanding of ornithischian phylogeny. *Convolosaurus marri* is clearly unique and defined by the apomorphies described above. *C*. *marri* overlaps temporally and geographically with *Tenontosaurus*, and the two are similar in morphology. However, in addition to the aforementioned apomorphies *C*. *marri* can be distinguished from *Tenontosaurus* by the presence of two supraorbital bones, narrow and elongate frontals, a predentary with a smooth oral margin, proximally positioned fourth trochanter on the femur, fully open posterior intercondylar groove of the femur, and the absence of an elongate tail. Furthermore, the manus phalangeal formula of *C*. *marri* (2-3-4-2-1) differs from that of *Tenontosaurus tilletti* (2-3-3-1?-1?; [26]); however, the count for *Tenontosaurus dossi* is unknown.

The nearly complete nature of the material recovered from Proctor Lake preserves informative characters shedding new light on their distributions in ornithischian phylogeny. For example, the presence of two supraorbital bones that extend across the entire orbit was thought to be a unique character of *Agilisaurus louderbacki* [23] and *Thescelosaurus neglectus* [21]. Previous studies indicated the presence of two supraorbital bones represented a primitive trait, recovering *Thescelosaurus neglectus* exclusive to a clade containing *H*. *foxii* and Iguanodontia [48]. However, this character is clearly present in *C*. *marri* and it was recovered more derived than *H*. *foxii*. This character is often not preserved, thus its presence in *C*. *marri* provides more information about its distribution in basal ornithischians.

## Conclusions

The Proctor Lake fossil locality contains a wealth of specimens providing not only nearly complete individual skeletons, but also insight into ontogeny and population structure. The femoral length distribution of 29 individuals from the Proctor Lake locality indicates a high mortality rate among the smallest and presumably youngest individuals. Clusters of individuals of varying sizes suggest individuals flocked together long after hatching perhaps for protection against predators. The specimens recovered from Proctor Lake reveal a new species of basal ornithopod with a unique set of both basal and derived characters. Characters including an expanded ischial foot, curved maxillary tooth roots, and opisthocoelus cervical vertebrae position *C*. *marri* in a clade exclusive of most basal ornithischians including *H*. *foxii* [18], but characters such as the presence of premaxillary teeth, shape of the frontals, and the position of the pterygoid wing on the quadrate position *C*. *marri* outside of Iguanodontia. Thus, this new species provides crucial information on the evolution of basal neornithischians.

## Supporting information

S1 Table Measurements of femora used in growth regression. (XLSX)

S2 Table Ontogenetic features. (XLSX)

S3 Table Scored character matrix. (XLSX)

S4 Table Characters rescored for *Tenontosaurus dossi*. (XLSX)

S1 Fig Map of selected major skeletal elements of 2DU fossil locality from Proctor Lake, Texas. (DOCX)

S2 Fig Thin Sections of *C*. *marri* femora. (DOCX)

S1 Text Character descriptions. (DOC)
