# Correction: A new basal ornithopod (Dinosauria: Ornithischia) from the Early Cretaceous of Texas

**DOI:** 10.1371/journal.pone.0217232

**Published:** 2019-08-06

**Authors:** Kate A. Andrzejewski, Dale A. Winkler, Louis L. Jacobs

Due to a technical error, S3 Table does not function properly. Please view a corrected S3 Table below.

## Supporting information

S3 Table Scored character matrix. (XLSX)
