# Supplementary material for: A new basal ornithopod (Dinosauria: Ornithischia) from the Early Cretaceous of Texas
Source: PLoS One. 2019 Mar 12;14(3):e0207935. doi: 10.1371/journal.pone.0207935 (PMC6413910; doi:10.1371/journal.pone.0207935)
Supplement: S1 Fig — (DOCX) [file pone.0207935.s005.docx]

Map of selected major skeletal elements of 2DU fossil locality from Proctor Lake, TX. Small and poorly preserved bones and those obscured by overlying elements were omitted for clarity. Femora used in Figure 28 highlighted in yellow. Map number and corresponding SMU catalog number and element description listed below. Scale bar equals 10 cm.


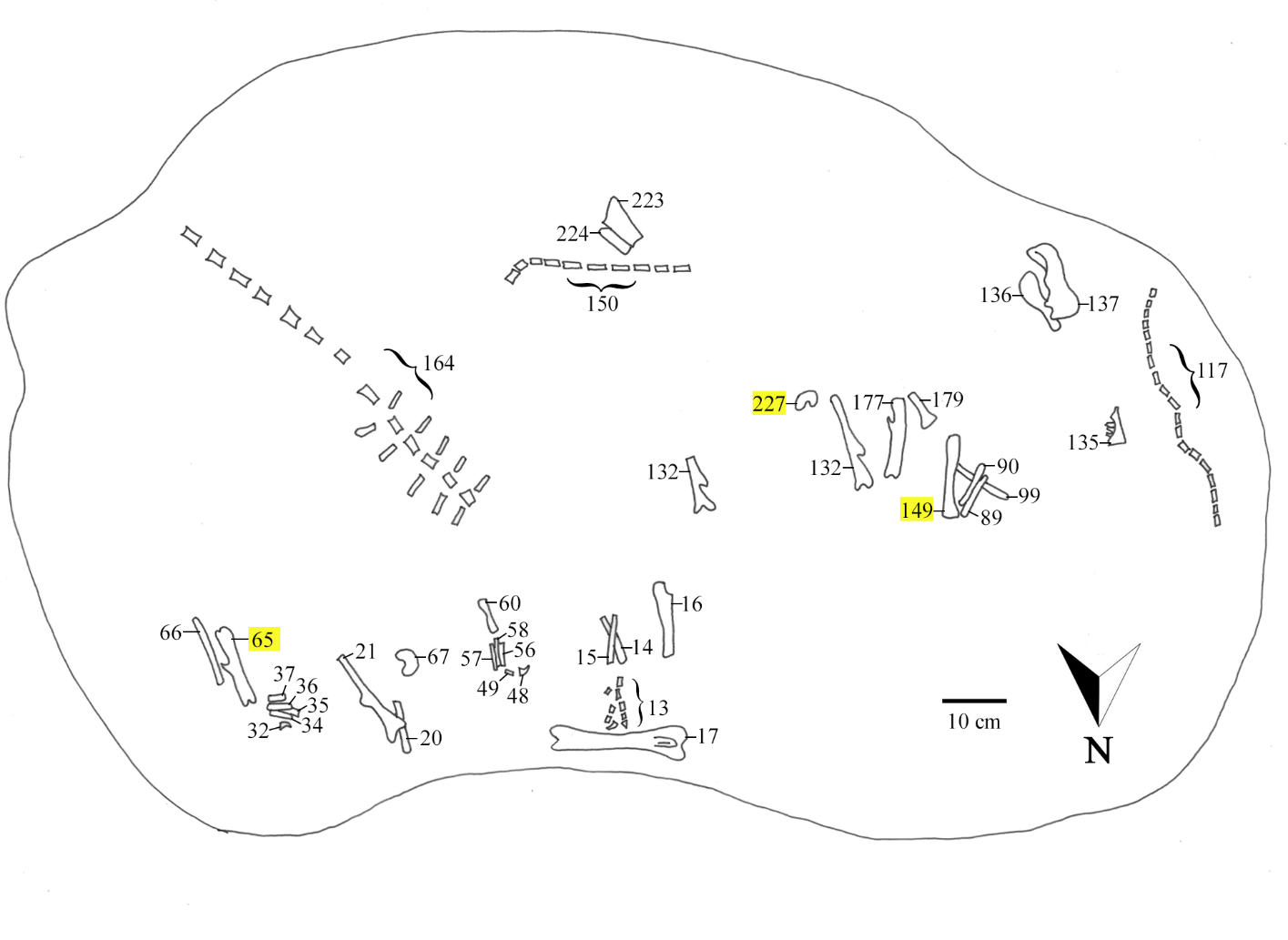


| **Map #** | **SMU Catalog** | **Element** |
| --- | --- | --- |
| 13 | 74083 | Left manus |
| 14 | 74083 | Left ulna |
| 15 | 74083 | Left radius |
| 16 | 74083 | Left humerus |
| 17 | 74086 | Left tibia |
| 20 | 74116 | Left humerus shaft |
| 21 | 74121 | Right ischium |
| 32 | 74085 | Left ungal pes |
| 34 | 74085 | Left metatarsal I |
| 35 | 74085 | Left metatarsal II |
| 36 | 74085 | Left metatarsal III |
| 37 | 74085 | Left metatarsal IV |
| 48 | 74091 | Left metatarsal I |
| 49 | 74091 | Left metatarsal II |
| 56 | 74091 | Left metatarsal IV |
| 57 | 74091 | Left phalanx pes |
| 58 | 74091 | Left ungal pes |
| 60 | 74120 | Left ischium |
| 65 | 74085 | Left femur |
| 66 | 74085 | Left fibula |
| 67 | 74098 | Right distal femur |
| 89 | 74089 | Right radius |
| 90 | 74088 | Right ulna |
| 99 | 74117 | Left fibula |
| 117 | 74084 | 19 articulated caudal vertebrae |
| 132 | 74119 | Right ischium, Left distal ischium |
| 135E | 74087 | Right dentary |
| 136 | 74122 | Right humerus |
| 137 | 74122 | Right scapula and coracoid |
| 149 | 74094 | Left femur |
| 150 | 74101 | 10 caudal vertebrae |
| 164 | 74130 | Associated caudal and sacral vertebrae |
| 177 | 74093 | Right femur |
| 223 | 74096 | Left tibia |
| 224 | 74096 | Left fibula |
| 227 | 74097 | Left distal femur |
