# Supplementary material for: A new basal ornithopod (Dinosauria: Ornithischia) from the Early Cretaceous of Texas
Source: PLoS One. 2019 Mar 12;14(3):e0207935. doi: 10.1371/journal.pone.0207935 (PMC6413910; doi:10.1371/journal.pone.0207935)
Supplement: S2 Fig — (DOCX) [file pone.0207935.s006.docx]

Thin sections of *C. marri* femora


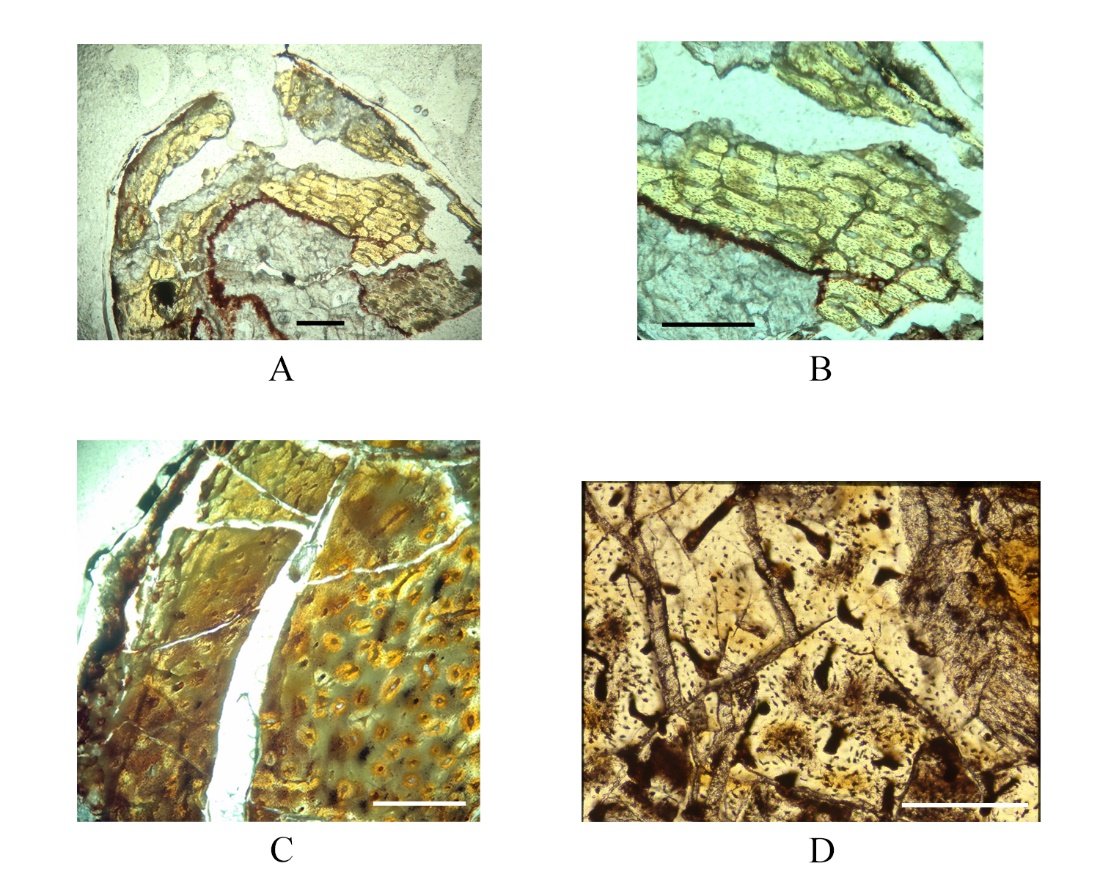


**Thin Sections of *C marri* femora**

(A) SMU 70447 femora at midshaft displaying highly vascularized lamellar bone with poorly developed primary osteons (B) SMU 70447 femora at midshaft detail of (A); (C) SMU 73569 femora at midshaft displaying primary and secondary osteons; (D) SMU 72834 femora at midshaft displaying primary and secondary osteons. Scale bar equals 1 mm.
